# DDX41 coordinates RNA splicing and transcriptional elongation to prevent DNA replication stress in hematopoietic cells

**DOI:** 10.1038/s41375-022-01708-9

**Published:** 2022-10-14

**Authors:** Satoru Shinriki, Mayumi Hirayama, Akiko Nagamachi, Akihiko Yokoyama, Takeshi Kawamura, Akinori Kanai, Hidehiko Kawai, Junichi Iwakiri, Rin Liu, Manabu Maeshiro, Saruul Tungalag, Masayoshi Tasaki, Mitsuharu Ueda, Kazuhito Tomizawa, Naoyuki Kataoka, Takashi Ideue, Yutaka Suzuki, Kiyoshi Asai, Tokio Tani, Toshiya Inaba, Hirotaka Matsui

**Affiliations:** 1 Department of Molecular Laboratory Medicine, Faculty of Life Sciences, Kumamoto University, Kumamoto, Japan; 2 Laboratory of Transcriptional Regulation in Leukemogenesis, International Research Center for Medical Sciences, Kumamoto University, Kumamoto, Japan; 3 Department of Molecular Oncology and Leukemia Program Project, Research Institute for Radiation Biology and Medicine, Hiroshima University, Hiroshima, Japan; 4 Tsuruoka Metabolomics Laboratory, National Cancer Center, Yamagata, Japan; 5 Isotope Science Center, The University of Tokyo, Tokyo, Japan; 6 Laboratory of Systems Genomics, Department of Computational Biology and Medical Sciences, Graduate School of Frontier Sciences, The University of Tokyo, Chiba, Japan; 7 Department of Nucleic Acids Biochemistry, Graduate School of Biomedical and Health Sciences, Hiroshima University, Hiroshima, Japan; 8 Laboratory of Genome Informatics, Department of Computational Biology and Medical Sciences, Graduate School of Frontier Sciences, The University of Tokyo, Chiba, Japan; 9 Department of Oral and Maxillofacial Surgery, Faculty of Life Sciences, Kumamoto University, Kumamoto, Japan; 10 Department of Biomedical Laboratory Sciences, Faculty of Life Sciences, Kumamoto University, Kumamoto, Japan; 11 Department of Neurology, Faculty of Life Sciences, Kumamoto University, Kumamoto, Japan; 12 Department of Molecular Physiology, Faculty of Life Sciences, Kumamoto University, Kumamoto, Japan; 13 Laboratory of Cellular Biochemistry, Department of Animal Resource Sciences, Graduate School of Agriculture and Life Sciences, The University of Tokyo, Tokyo, Japan; 14 Department of Biological Sciences, Faculty of Advanced Science and Technology, Kumamoto University, Kumamoto, Japan

**Keywords:** Haematological cancer, Haematological cancer

## Abstract

Myeloid malignancies with *DDX41* mutations are often associated with bone marrow failure and cytopenia before overt disease manifestation. However, the mechanisms underlying these specific conditions remain elusive. Here, we demonstrate that loss of DDX41 function impairs efficient RNA splicing, resulting in DNA replication stress with excess R-loop formation. Mechanistically, DDX41 binds to the 5′ splice site (5′SS) of coding RNA and coordinates RNA splicing and transcriptional elongation; loss of DDX41 prevents splicing-coupled transient pausing of RNA polymerase II at 5ʹSS, causing aberrant R-loop formation and transcription-replication collisions. Although the degree of DNA replication stress acquired in S phase is small, cells undergo mitosis with under-replicated DNA being remained, resulting in micronuclei formation and significant DNA damage, thus leading to impaired cell proliferation and genomic instability. These processes may be responsible for disease phenotypes associated with *DDX41* mutations.

## Introduction

*DDX41* mutation occurs in various hematopoietic malignancies, most frequently in acute myeloid leukemia (AML) and myelodysplastic syndromes (MDS) [1–4]. *DDX41* encodes a DEAD-box-type RNA helicase that mainly localizes in the nucleus. Proposed biological functions of nuclear DDX41 include R-loop resolution [5, 6], small nucleolar RNA processing [7] and ribosomal RNA (rRNA) processing [8]. DDX41 was also found in the spliceosome [9–12]. Notably, it has been shown that some individuals with a germline *DDX41* variant in one allele later develop hematopoietic malignancies by acquiring a somatic mutation in the other allele. Most germline *DDX41* variants are frameshift, nonsense, or missense mutations that occur in the entire coding region without any hotspots, which suggests that these germline variants lose expression or function. On the other hand, somatic mutations are highly concentrated in the R525H mutation located within the helicase domain where DDX41 interacts with ATP. Indeed, our previous study revealed reduced ATPase activity for the helicase domain with the R525H mutation [8]. In addition, compound heterozygous mutations combining a germline variant and the somatic R525H mutation are observed in human AML/MDS, whereas homozygous *Ddx41* knockout mice are lethal [13]. Collectively, somatic mutants are considered to be functionally hypomorphic, and the acquisition of somatic mutations to cells with germline variants would be expected to further reduce the activity of DDX41 to the extent that it is not completely lost. The average age of disease onset for patients with a germline *DDX41* variant is the 60 s, which is comparable to that of patients without the variant [2–4, 14, 15]. However, individuals with a heterozygous germline *DDX41* variant present unexplained cytopenia of one or more hematopoietic lineages before the development of hematopoietic malignancies at the rate of 40–66% [1, 16].

Patients with *DDX41* mutations often exhibit hypoplastic bone marrow, which is relatively characteristic of MDS/AML with this mutation. In addition, *DDX41* mutations in MDS/AML are not necessarily mutually exclusive with those in genes encoding typical MDS-related RNA splicing factors [17–19], suggesting unique pathological implications of *DDX41* mutations somewhat different from those of other MDS-related RNA splicing factors.

Here, we demonstrate that DDX41 mainly binds to 5ʹ splice sites (SS) of coding RNA and is involved in the formation of activated spliceosomes. DDX41 was responsible for interaction between the spliceosome and transcriptional elongation complex, thereby coordinating the two distinct machineries. Suppression of DDX41 function thus caused altered transcriptional elongation and R-loop accumulation, which led to mild DNA replication stress in S phase. Consequently, cells undergo mitosis without resolving this replication stress, leading to impaired cell proliferation and genomic instability.

## Results

### DDX41 is an RNA splicing factor that binds mainly to the 5ʹSS

To investigate the role of DDX41 in RNA biogenesis, we analyzed RNAs with which DDX41 may interact, by performing ultraviolet (UV) crosslinking, immunoprecipitation (IP), and sequencing (CLIP-seq) [20, 21]. We crosslinked K562 cells expressing Myc-tagged wild-type (WT) or R525H mutant DDX41, most frequent somatic mutant found in myeloid malignancies, via UV light, and sequenced RNAs co-precipitated with Myc-DDX41 (Supplementary Fig. S1A).

**Fig. 1:**
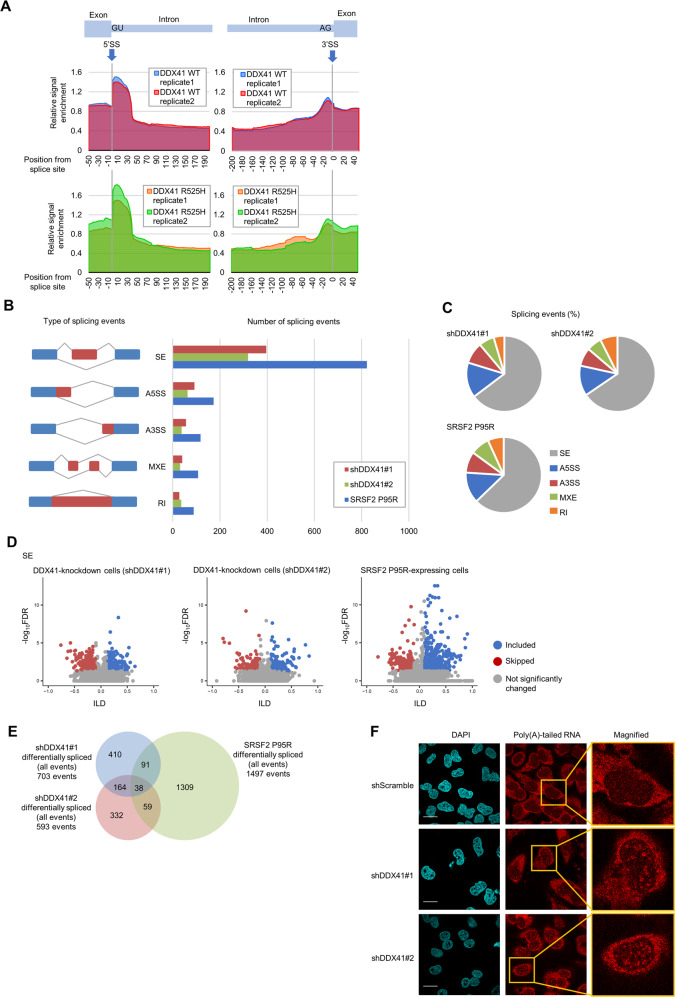
DDX41 is involved in RNA splicing by binding to 5’SS but does not play a major role in SS recognition. **A** Relative CLIP-seq signals at 5ʹSS and 3ʹSS on coding RNA. Vertical axis: ratio of CLIP sample signal divided by that of input RNA from same cells. Blue and red lines in top panels indicate relative signal enrichment of CLIP reads from cells expressing Myc-tagged WT DDX41; green and orange lines in bottom panels indicate reads from cells expressing Myc-tagged R525H mutant DDX41. **B** Quantification of RNA splicing changes in K562 cells expressing shDDX41#1, shDDX41#2, or SRSF2 P95R. We placed splicing events into groups according to rMATS: (1) skipped exon (SE), (2) alternative 5ʹSS (A5SS), (3) alternative 3ʹSS (A3SS), (4) mutually exclusive exons (MXE), and (5) retained intron (RI). Cumulative number of events in each cell group with an inclusion level difference (ILD) > 0.1 or <−0.1 and a false discovery rate (FDR) <0.05 are shown. **C** Distribution of RNA splicing events in K562 cells expressing shDDX41#1, shDDX41#2, or SRSF2 P95R compared with control K562 cells. Splicing events were categorized as in **B**. **D** Changes in RNA splicing events for SE in DDX41-knockdown cells and SRSF2 P95R-expressing cells. We included splicing events with 10% minimum change of absolute percent spliced-in index (PSI, which indicates rate of incorporation of specific exon into transcript of a gene) (delta PSI ≥ 0.1) and average reads ≥5; those with FDR < 0.05 with ILD < 0.1 or >0.1 in each group were considered significant and plotted with red or blue dots, respectively. Gray dots are not significant. **E** Overlap of RNA splicing events among DDX41-knockdown cells and SRSF2 P95R-expressing cells. All significant RNA splicing events (SE, MXE, RI, A5SS, and A3SS) in each cell type were summed, and event overlap among DDX41-knockdown cells and SRSF2 P95R-expressing cells is shown. **F** Subcellular distribution of poly(A)-tailed RNA in DDX41-knockdown cells. Scale bars: 20 μm.

First, we mapped the sequenced reads to ribosomal DNA (rDNA) and found that 60.0–65.1% CLIP-seq reads were uniquely mapped to rDNA, especially to the 18S and 28S rRNA regions, after removing duplicate reads (Supplementary Fig. S1B, C). Therefore, DDX41 likely interacts primarily with mature rRNA and may play a role in ribosome biogenesis, as we suggested previously [8]. We then mapped the remaining sequence reads to human genome and found that 24.7–28.7% CLIP-seq reads per sample were uniquely mapped to coding genes (Supplementary Fig. S1B), indicating that DDX41 also binds to coding RNA. DDX41 preferentially bound to 5ʹSS, and somewhat less to 3ʹSS, on coding RNAs (Fig. 1A). Mutant R525H DDX41 also bound to rRNA and coding RNA in the same manner as did WT DDX41 (Fig. 1A and Supplementary Fig. S1C), which suggests that R525H mutant retains comparable RNA-binding activity to WT DDX41, although the mutant has reduced ATPase activity [8].

Because DDX41 bound to or near SS on coding RNAs, we analyzed RNA splicing changes using rMATS [22] by comparing RNA sequencing (RNA-seq) data for K562 cells with suppressed DDX41 expression (DDX41-knockdown cells) by DDX41-specific short hairpin RNAs (shRNA) (shDDX41#1 or shDDX41#2) with data for control cells expressing scrambled shRNA (shScramble). As a reference, we included RNA-seq data for cells expressing SRSF2 P95R, one of the RNA splicing-related mutants found in MDS, in the analysis. Similar trends of RNA splicing changes were observed in both DDX41-knockdown cells and SRSF2 P95R-expressing cells (Fig. 1B, C). However, RNA splicing changes in DDX41-knockdown cells were less frequent than those of SRSF2 P95R-expressing cells (Fig. 1B) and were either increased or decreased for each event type, with no particular trend toward either direction (Fig. 1D and Supplementary Fig. S1D), which differed from those of SRSF2 P95R-expressing cells in which skipped exon (SE) was relatively suppressed [23].

Figure 1E shows that only 202 of 703 and 593 differentially spliced events in shDDX41#1- and shDDX41#2-expressing cells, respectively, overlapped. There was also little overlap when aggregated across all shared events that could affect the same exons (Supplementary Fig. S1E). No relevant sequence features existed in differentially spliced exons and 5’SS and 3’SS (Supplementary Fig. S1F, G), although we found that the DDX41 deficiency induced splicing changes in genes partially similar to those seen in myeloid malignancies with mutations in RNA splicing factors accompanied by impaired mRNA production (Supplementary Fig. S1H, I, J, K).

These results suggest that DDX41 may be involved in RNA splicing and mRNA synthesis by interacting with 5’SS, but play smaller roles in determining RNA splicing position and inclusion/exclusion of exons. Nevertheless, RNA fluorescence in situ hybridization with oligo(dT) probes demonstrated speckle-like signals in DDX41-knockdown cell nuclei (Fig. 1F), suggesting that the loss of DDX41 caused impaired maturation or export of mRNA due to a defect in mRNA synthesis.

### DDX41 interacts with activated spliceosomes

**Fig. 2:**
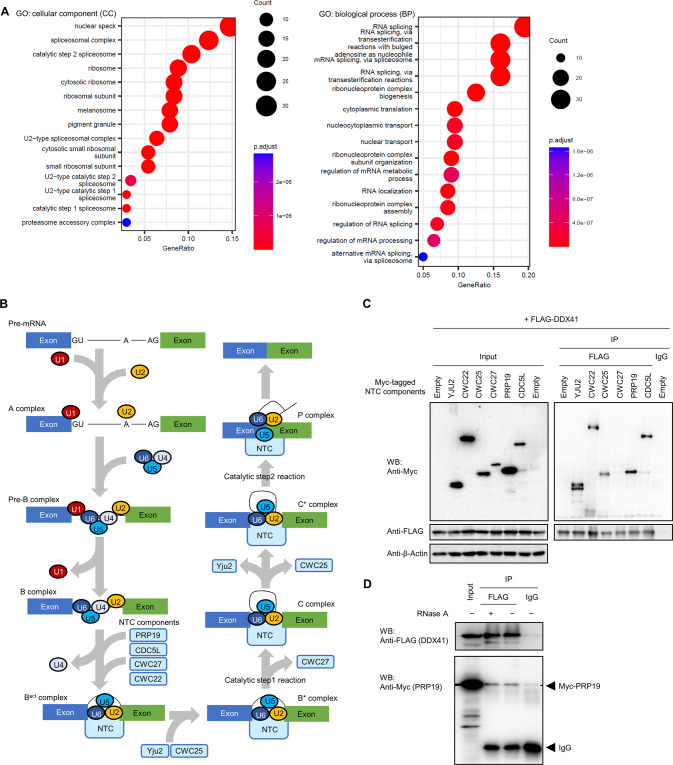
Interaction of DDX41 with RNA splicing-related proteins. **A** Major GOs of proteins interacted with FLAG-DDX41. Nuclear proteins immunoprecipitated with FLAG-DDX41 were categorized via GO analysis. Top 15 GO terms for CC (cellular component) and BP (biological process) categories are shown, with the number of genes indicated by circle sizes and adjusted *p* values indicated by red to blue colors. **B** Schematic diagram showing NTC involvement in RNA splicing. The factors within NTC are incorporated into or excluded from the complex depending on the splicing steps, in which core NTC components (PRP19 and CDC5L) occur throughout NTC after incorporation of the complex into the spliceosome; CWC25 and Yju2 are incorporated before the B^*^ complex and excluded before the C^*^ complex, and CWC27 is excluded before the C complex. **C** Interaction of DDX41 with RNA splicing process-specific components in the NTC. Myc-tagged NTC components (Yju2, CWC22, CWC25, CWC27, PRP19, and CDC5L) were expressed with FLAG-tagged DDX41 in HEK293FT cells, and DDX41-interacting proteins were immunoprecipitated with an anti-FLAG antibody. Precipitated proteins were probed with anti-FLAG, anti-Myc, or anti-β-Actin antibody. Left and right panels indicate input and immunoprecipitated samples, respectively. **D** Non-RNA-mediated interaction of DDX41 with PRP19. FLAG-tagged DDX41 and Myc-tagged PRP19 were expressed in HEK293FT cells, and FLAG-DDX41 was immunoprecipitated with anti-FLAG antibody. Precipitated samples were then treated with 20 μg/ml RNase A for 30 min at 37 °C before being probed with anti-FLAG or anti-Myc antibody.

To further investigate the roles of DDX41 in RNA splicing, we analyzed proteins with which DDX41 may interact. FLAG-tagged DDX41 was immunoprecipitated with its interacting proteins from the nuclear fraction of FLAG-DDX41-expressing K562 cells, and we analyzed them by mass spectrometry. Gene ontology (GO) analysis of proteins specifically interacted with FLAG-DDX41 revealed that DDX41 had a high likelihood of interacting with proteins related to RNA splicing (Fig. 2A and Supplementary Fig. S2), including catalytic steps 1 and 2 spliceosomes. Because these spliceosomes include several components of the NineTeen complex (NTC), we investigated interactions of DDX41 with the NTC. In RNA splicing, the NTC with PRP19 as a core component joins the spliceosome together with U4/U6.U5 tri-snRNP and plays essential roles in spliceosome activation and reorganization by exchanging factors in the complex (Fig. 2B) [24]. NTC also promotes transcriptional elongation by interacting with an RNA polymerase II (Pol II)-containing complex [25, 26]. We found that DDX41 interacted with PRP19 and CDC5L, which are core components of NTC, and Yju2 (CCDC94), CWC22, and CWC25, which are NTC-related factors, but DDX41 interacted very weakly with CWC27 (Fig. 2C). Interaction between DDX41 and PRP19 was likely not mediated by RNA, because RNase A treatment did not impair the interaction (Fig. 2D). Because CWC27 dissociate from spliceosome during conversion from B* to C complex [27], and CWC25 and Yju2 from C to C* complex [24] (Fig. 2B), the spliceosome with which DDX41 mainly interacted appeared to be C complex that has completed SS recognition. This result agrees with papers reporting that DDX41 occurred in the C complex containing activated spliceosome [9, 10, 24, 28].

### DDX41 prevents impaired DNA replication and mitotic abnormalities

**Fig. 3:**
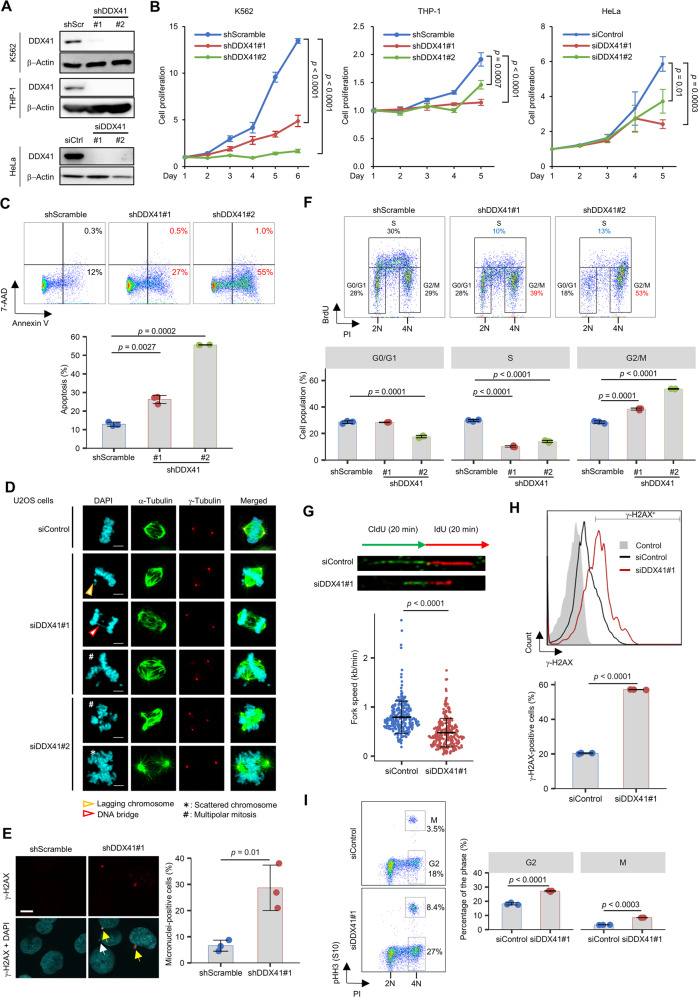
DDX41-knockdown induces impaired DNA replication and abnormal mitosis. **A** Inhibition of DDX41 expression by siRNA/shRNA against DDX41. shScr, shScramble; siCtrl, siControl. **B** Suppression of cell growth by DDX41 knockdown. Cells were transfected with the indicated shRNAs (K562 and THP-1 cells) or siRNAs (HeLa cells). Day 1 means day 4 after lentivirus infection (K562 and THP-1 cells; *n* = 4) or the day of siRNA transfection (HeLa cells; *n* = 3). Values are means ± SD; two-tailed unpaired Student’s *t* test. **C** Induction of apoptosis by DDX41 knockdown. (Upper) Representative images of flow cytometric analysis of K562 cell apoptosis. (Lower) Bars indicate means; error bars, SDs of triplicate samples; two-tailed Welch’s *t* test. **D** Abnormal mitosis after DDX41 knockdown in U2OS cells. Scale bars: 5 μm. **E** Micronucleus formation by DDX41 knockdown in K562 cells. Percent micronucleus-positive cells in shScramble (*n* = 176, 194 and 233 cells) and shDDX41#1 (*n* = 84, 100 and 115 cells) groups were analyzed 3 days after lentivirus infection. (Left) Yellow and white arrows indicate micronuclei positive and negative for γ-H2AX, respectively. Scale bar: 10 μm. (Right) Bars indicate means; error bars, SD of triplicate samples; two-tailed unpaired Student’s *t* test. **F** Inhibition of DNA replication by DDX41 knockdown in K562 cells. (Upper) Representative images of flow cytometric analysis of BrdU incorporation. (Lower) Bars indicate means; error bars, SD of triplicate samples; two-tailed Welch’s *t* test. **G** Slowed replication fork progression by DDX41 knockdown in HeLa cells. (Upper) Dual labeling with DNA analogs and representative images of DNA fibers. Thymidine analogues were visualized via immunofluorescence (CldU, green; IdU, red). (Lower) Fork progression speed calculated for each sample. Bars indicate means ± SD; *n* = 269 and 271 for siControl and siDDX41#1, respectively; two-tailed Welch’s *t* test. **H** Induction of γ-H2AX signals by DDX41 knockdown in HeLa cells. Three days after siRNA transfection, flow cytometry analysis was performed. (Upper) Histogram of γ-H2AX levels. (Lower) Percent γ-H2AX-positive cells in each cell group. Bars indicate means; error bars, SD of triplicate samples; two-tailed Welch’s *t* test. **I** Cell cycle changes by DDX41 knockdown in HeLa cells. Three days after siRNA transfection, flow cytometry analysis was performed. (Left) Representative flow cytometry plots of cells stained with anti-phosphorylated histone H3 (pHH3) antibody and Propidium Iodide (PI). (Right) Percent cells at G2 and M phases. Bars indicate means; error bars, SD of triplicate samples; two-tailed Welch’s *t* test.

To clarify the biological significance of DDX41, we suppressed its expression in different cell lines (Fig. 3A). DDX41 knockdown with small interfering RNAs (siRNAs) (siDDX41#1 and siDDX41#2 for HeLa) or shRNAs (shDDX41#1 and shDDX41#2 for K562 and THP-1) significantly suppressed cell proliferation in all cell lines (Fig. 3B). We also observed increased apoptosis for DDX41-knockdown cells (Fig. 3C). These observations are consistent with recent reports including non-mammalian studies [7, 29, 30], although a previous report suggested a conflicting phenotype [31].

Next, we examined cell cycle progression using U2OS cells stably expressing GFP-tagged histone H2B. DDX41-knockdown cells had a longer mitotic period, with abnormal mitosis and post-mitotic nuclear morphologies (Supplementary Fig. S3A and Supplementary Movies 1 to 3). The cells frequently manifested multipolar centrosomes and scattered chromosomes in metaphase and lagging chromosome and DNA bridges in anaphase (Fig. 3D), which were also the case in HeLa cells and even in non-cancerous ARPE-19 epithelial cells (Supplementary Fig. S3B). γ-H2AX-positive micronuclei, a hallmark of genomic instability, were frequently found in DDX41-knockdown K562 cells (Fig. 3E and Supplementary Fig. S3C).

We observed G2/M arrest and impaired DNA replication in DDX41-knockdown cells, as indicated by the increase in cells with double amount of DNA (4N) and reduced bromodeoxyuridine (BrdU) incorporation (Fig. 3F). Slowed replication fork progression was also observed in a DNA fiber assay (Fig. 3G), accompanied by an enhanced γ-H2AX signal (Fig. 3H), which is consistent with a previous report describing the involvement of RNA processing factors in DNA damage response [32]. Impaired DNA replication here was not severe enough to induce S-phase arrest (Fig. 3F). However, we noted that the G2 phase population substantially contributed to G2 and M phase accumulation on day 3 of siDDX41 transfection in additional analyses with anti-phosphorylated histone H3 (pHH3) antibody for mitotic cells (Fig. 3I). Thus, problems with DNA replication during S phase, even though they were unnoticeable, may trigger significant cell cycle changes and abnormal cellular morphology.

### Functional inhibition of DDX41 induces mild DNA replication stress that leads to delayed G2-M transition

**Fig. 4:**
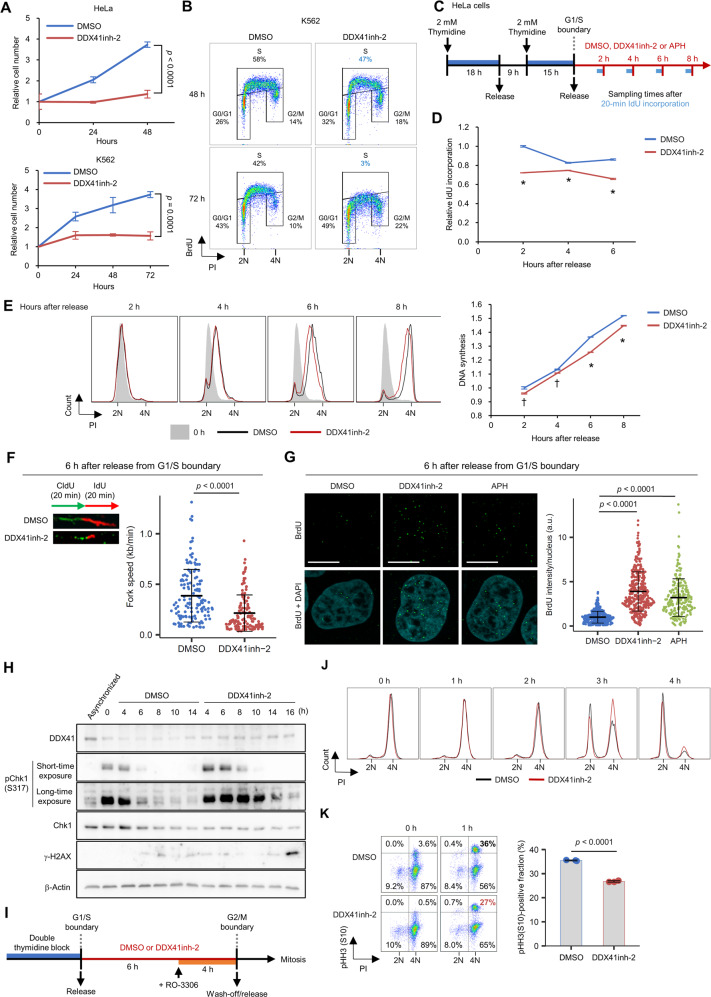
DDX41 inhibition induces mild DNA replication stress in S phase. **A** Suppression of HeLa and K562 cell proliferation by DDX41 inhibition (50 μM DDX41inh-2 treatment). Cell number was counted by using trypan blue. Values are means ± SD of triplicate samples; two-tailed unpaired Student’s *t* test. **B** Reduced BrdU incorporation by 50 μM DDX41inh-2 treatment in HeLa cells. **C** Schematic diagram of cell cycle synchronization, drug treatment, and IdU incorporation in HeLa cells. **D** Reduced IdU incorporation in S phase by 50 μM DDX41inh-2 treatment in HeLa cells. *p < 0.0001, two-tailed unpaired Student’s *t* test. Results of the 8-h treatment are not shown because S-G2 transition occurred (see Supplementary Fig. S4B). **E** Delayed S-phase progression in HeLa cells by 50 μM DDX41inh-2 treatment. (Left) Representative histograms. (Right) DNA synthesis rate as estimated by median fluorescence intensity (MFI) of PI. Values are means ± SD of triplicate samples; *p < 0.0001; ^†^*p* < 0.005, two-tailed unpaired Student’s *t* test. **F** Slowed replication fork progression by DDX41 inhibition. (Left) Experimental scheme of dual labeling with DNA analogs and representative images of DNA fibers. Thymidine analogues were visualized via immunofluorescence (CldU, green; IdU, red). (Right) Fork progression speed was calculated for each sample. Bars represent means ± SD; *n* = 139 and 134 for DMSO and DDX41inh-2 group, respectively; two-tailed Welch’s *t* test. **G** Increase in single-s*t*randed DNA by treatment with 50 μM DDX41inh-2 or 10 nM APH. Scale bars: 10 μm. Bars represent means ± SD; *n* = 400, 380 and 222 nuclei for DMSO, DDX41inh-2, and APH, respectively; two-tailed Welch’s *t* test. **H** Increase in DNA damage-related signals by DDX41 inhibition in HeLa cells. Protein extracts obtained from cell cycle-synchronized HeLa cells were probed with antibodies indicated at left. The time indicated are the hours after release from G1/S arrest. **I** Schematic diagram of mitosis assessment in HeLa cells after 50 μM DDX41inh-2 treatment during S phase. RO-3306, a CDK1 inhibitor, was used to induce G2 arrest. **J**, **K** Delayed mitosis in HeLa cells after DDX41 inhibition in S phase. HeLa cells were treated as indicated in **I**. Flow cytometry analysis of cell cycle change (**J**) and proportion of cells at M phase (**K**). Cells at G2 or M were determined as those negative or positive for pHH3 with PI signal corresponding to 4N, respectively. Right panel in **K** indicates proportion of pHH3-positive M phase cells 1 h after RO-3306 removal. Bars indicate means, error bars indicate SD of triplicate samples; two-tailed Welch’s *t* test.

To better understand the roles of DDX41, we analyzed cell phenotypes after acute inhibition of DDX41, by using recently identified DDX41 inhibitors, [(4*E*)-*N*-hydroxy-1-phenyl-2,3-dihydroquinolin-4(1*H*)-imine] and [4-amino-6-((2-amino-1,6-dimethylpyrimidin-4(1*H*)-ylidene)amino)-1-methyl-2-phenylquinolinium chloride hydrochloride] (DDX41inh-1 and DDX41inh-2, respectively), which were demonstrated to inhibit ATPase activity of DDX41 [33]. These inhibitors suppressed cell growth of HeLa and K562 in a concentration-dependent manner (Supplementary Fig. S4A). Because DDX41inh-2 showed a greater anti-proliferative effect than DDX41inh-1, as reflected by their inhibitory effects on ATPase activity of DDX41 [33], we used DDX41inh-2 for subsequent analyses. We confirmed that DDX41inh-2 suppressed proliferation of both cell lines after 48–72 h of culture in a time-dependent manner (Fig. 4A). Given that DDX41 exerts RNA helicase activities in an ATPase-dependent manner [34], the helicase activity of DDX41 likely plays an important role in cell proliferation.

In accord with the knockdown experiments, DDX41inh-2 inhibited BrdU incorporation (Fig. 4B). However, the reduction in BrdU incorporation was modest at 48 h after addition of the inhibitor, when cell growth was markedly inhibited, and as long as 72 h was needed before a marked decrease in BrdU incorporation. This finding supports our hypothesis that problems resulting from DNA replication defects, rather than impaired replication itself, were responsible for the marked cell cycle changes.

Next, we investigated DNA replication in the presence of DDX41inh-2 after release from the G1/S boundary in cell cycle-synchronized HeLa cells (Fig. 4C). DDX41 inhibition subtly reduced 5-iodo-2ʹ-deoxyuridine (IdU) incorporation (Fig. 4D and Supplementary Fig. S4B), thereby slightly slowing DNA synthesis and replication fork progression through S phase (Fig. 4E, F).

These cellular changes associated with DDX41 inhibition were similar to mild replication stress observed with low concentrations of aphidicolin (APH), a DNA polymerase α inhibitor (Supplementary Fig. S4C, D) [35, 36]; APH treatment at 10 nM induced a slight delay in S-phase progression, as did the DDX41 inhibitor (Fig. 4E and Supplementary Fig. S4E).

We found that DDX41 inhibition for 6 h during S phase increased single-stranded DNA to the same extent as did 10 nM APH (Fig. 4G). Enhanced and prolonged Chk1 activation in S phase was also seen in DDX41inh-2-treated cells (Fig. 4H). These data indicated that mild DNA replication stress was induced by DDX41 inhibition. Nevertheless, an apparent increase in γ-H2AX signal was not observed until cells completed mitosis (Fig. 4H and Supplementary Fig. S4F), suggesting the requirement for mitosis in DDX41-inhibited cells to manifest marked DNA damage.

We also tested how DNA replication stress by DDX41 inhibition affected mitosis by arresting synchronized cells at G2 phase in the presence of DDX41inh-2, followed by its wash-off (Fig. 4I and Supplementary Fig. S4G). Cell cycle analysis showed that cells pretreated with DDX41inh-2 in S/G2 had a delayed return from 4N to 2N (Fig. 4J) and delayed G2-M transition (Fig. 4K), whereas DDX41 inhibition initiated from the end of G2 did not cause these delays (Supplementary Fig. S4H, I). These data indicated that mild DNA replication stress induced by DDX41 inhibition delayed G2-M transition, consistent with our findings showing G2 phase accumulation for asynchronized DDX41-knockdown cells (Fig. 3I).

### DNA replication stress by DDX41 inhibition induces mitotic abnormalities and leaves DNA damage in post-mitotic cells

**Fig. 5:**
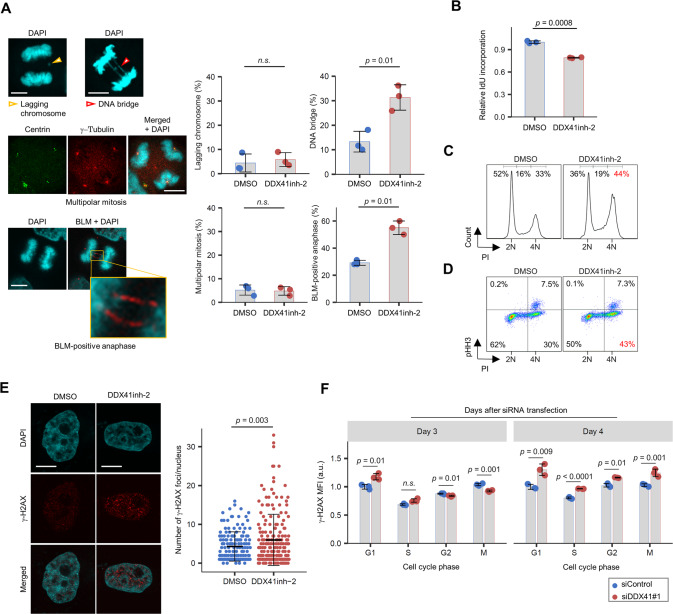
Mild replication stress by DDX41 inhibition triggers mitotic abnormalities and affects cell cycle progression of daughter cells. **A** Increased DNA bridges and ultrafine DNA bridges in mitotic HeLa cells treated with DDX41inh-2 during S phase. See Supplementary Fig. S5A for schematic. (Left) Representative images of abnormal mitosis. (Right) Quantitative result of abnormal mitosis. Bars indicate means; error bars, SD of triplicate samples; two-tailed Welch’s *t* test. *n.s*., not significant. Scale bars: 10 μm. **B** Reduced IdU incorporation in cells that had been treated with DDX41inh-2 in S phase and had undergone mitosis. See Supplementary Fig. S5A for schematic. Bars indicate means; error bars, SD of triplicate samples; two-tailed Welch’s *t* test. **C**, **D** Cell cycle arrest at G2 phase in HeLa cells that had been treated with DDX41inh-2 in S phase and had undergone mitosis. Cells were treated as in Fig. 4I. Cell cycle status 18 h after removal of DDX41inh-2 and RO-3306 was identified by PI staining (**C**). Cells were double-stained with PI and anti-pHH3 antibody to distinguish mitotic cells from cells at G2 (**D**). **E** Increase in γ-H2AX foci in G1 HeLa cells that had been treated with DDX41inh-2 in S phase and had undergone mitosis. Cells were stained with anti-γ-H2AX antibody and DAPI. See Supplementary Fig. S5B for schematic. Bars represent means ± SD; *n* = 151 and 195 for DMSO and DDX41inh-2, respectively; two-tailed Welch’s *t* test. Scale bars: 10 μm. **F** Increase in γ-H2AX signals primarily occurred at G1 in HeLa cells after DDX41 knockdown. Cells were stained with anti-γ-H2AX and anti-pHH3 antibodies and PI. MFI of γ-H2AX in each cell cycle phase was analyzed with flow cytometry. Bars indicate means; error bars, SD of triplicate samples; two-tailed Student’s *t* test.

We then analyzed how DNA replication stress by DDX41 inhibition affected mitosis. We analyzed mitosis after 8 h of exposure to DDX41inh-2 during S phase without CDK1 inhibition by RO-3306 (Supplementary Fig. S5A). Although the extent of lagging chromosome and multipolar mitosis was comparable for DDX41inh-2-treated cells and DMSO-treated cells, DDX41inh-2 treatment increased mitotic DNA bridges and ultrafine bridges positive for Blooms syndrome protein (BLM), which strongly suggested an increase in under-replicated DNA in S phase (Fig. 5A) [37].

DDX41 inhibition during S phase led to reduced IdU incorporation (Fig. 5B and Supplementary Fig. S5A) and subsequent G2 arrest in daughter cells (Fig. 5C, D), similar to the results in asynchronized DDX41-knockdown cells (Fig. 3F). We also found that DDX41 inhibition in S phase increased the number of nuclear γ-H2AX foci in G1 daughter cells (Fig. 5E and Supplementary Fig. S5B). In DDX41-knockdown cells, modest γ-H2AX accumulation first occurred in G1 (Fig. 5F, Day 3), before accumulation expanded to all cell cycle phases (Fig. 5F, Day 4). These observations suggest that modest DNA replication stress acquired during S phase by DDX41 inhibition persists and that cells enter mitosis in an under-replicated state; interphase checkpoint mechanisms would overlook replication stress caused by DDX41 inhibition, which would let cells enter mitosis without sufficient repair of DNA lesions.

### DDX41 protects DNA from R-loop formation

We postulated that impaired RNA splicing caused by DDX41 deficiency induced formation of an R-loop, a genomic structure that consists of a DNA:RNA hybrid and displaced single-stranded DNA, which in turn causes DNA replication stress [38].

**Fig. 6:**
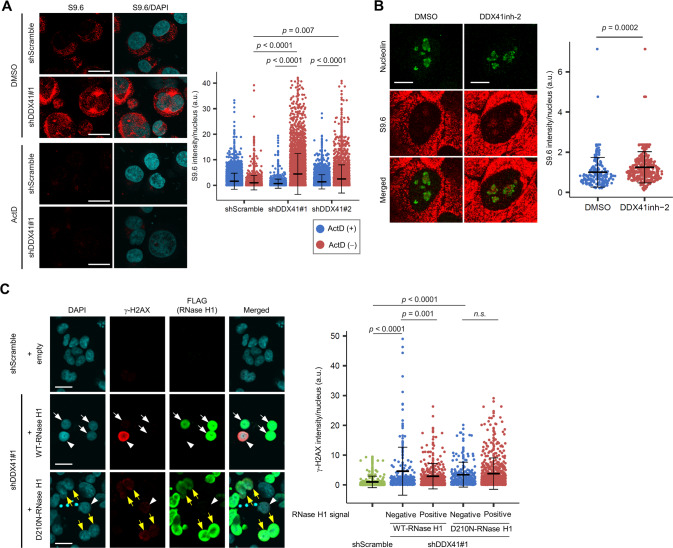
Loss of expression or function of DDX41 induces R-loop accumulation. **A** Increase in nuclear S9.6 signals by DDX41 knockdown. K562 cells infected with lentivirus expressing shDDX41#1, shDDX41#2, or shScramble were treated with ActD or DMSO for 6 h. Cells were stained with S9.6 antibody and DAPI. (Left) Representative immunofluorescence images. Scale bars: 20 μm. (Right) Nuclear S9.6 signal intensity. Bars represent means ± SD; *n* = 2645, 1628, 1490, 2357, 2095 and 2401 nuclei for shScramble/ActD^+^, shScramble/ActD^−^, shDDX41#1/ActD^+^, shDDX41#1/ActD^−^, shDDX41#2/ActD^+^, and shDDX41#2/ActD^−^, respectively; two-tailed Welch’s *t* test. **B** Increase in nuclear S9.6 signals in S phase by DDX41 inhibition. HeLa cells were treated with 50 μM DDX41inh-2 or DMSO as in Fig. 4C. Six hours after release from the G1/S boundary, cells were stained with S9.6 antibody and DAPI. S9.6 signals from nucleoli were subtracted, and signals in nucleoplasm were quantitatively measured. (Left) Representative immunofluorescence images. Scale bars: 10 μm. (Right) Nuclear S9.6 signal intensity. Bars represent means ± SD; *n* = 187 and 407 for DMSO and DDX41inh-2, respectively; two-tailed Welch’s *t* test. **C** Reduced γ-H2AX signals by enforced expression of RNase H1 in DDX41-knockdown cells. K562 cells were infected with lentivirus expressing shDDX41#1, shDDX41#2, or shScramble. Five days later, cells were transfected with FLAG-tagged nuclear-localizing RNase H1-expressing vector or an empty vector; 2 days later, cells were stained with anti-γ-H2AX and anti-FLAG antibodies and DAPI. (Left) Representative immunofluorescence images. White arrowheads, white arrows, and yellow arrows indicate γ-H2AX^+^/RNase H1^−^ cells, WT-RNase H1^+^ cells, and γ-H2AX^+^/D210N-RNase H1^+^ cells, respectively. Scale bars: 20 μm. (Right) γ-H2AX signal intensity for cells negative and positive for RNase H1 signal. Bars represent means ± SD; *n* = 394, 214, 342, 232 and 612 for shScramble, shDDX41#1/WT-RNase H1^−^, shDDX41#1/RNase H1^+^, shDDX41#1/D210N-RNase H1^−^, and shDDX41#1/D210N-RNase H1^+^, respectively; two-tailed Welch’s *t* test.

Immunofluorescence staining of cells with S9.6 antibody, which is used to detect R-loops [39], showed increased nuclear S9.6 signals in DDX41-knockdown cells (Fig. 6A). Treatment with actinomycin D (ActD) inhibited this S9.6 signal induction (Fig. 6A), which confirmed that accumulation of R-loops by DDX41 knockdown depended on transcription. Increased nuclear S9.6 signals by DDX41inh-2 were also noted in the first S phase after G1 arrest (Fig. 6B). In support of our hypothesis, overexpression of RNase H1 modified to preferentially localize to the nucleus [40] attenuated γ-H2AX signal induction in DDX41-knockdown cells (Fig. 6C). These data indicated that R-loops aberrantly formed in a transcription-dependent manner were responsible for the DNA damage caused by loss of DDX41 function.

Given this, we immunoprecipitated R-loops from HEK293FT cells expressing Myc-tagged RNase H1 mutant (D210N) that lacks enzymatic activity but retains R-loop-binding capacity (Supplementary Fig. S6A, B). Identified 431 R-loop-interacting proteins, of which 26–53% overlapped with those identified in other studies [5, 41, 42], included certain RNA processing factors and Pol II, confirming efficient R-loop recovery (Supplementary Fig. S6B  and Supplementary Table S1). However, DDX41 was not identified in the co-precipitates in our assay (Supplementary Fig. S6B and Supplementary Table S1), suggesting that the primary cause of R-loop accumulation by loss of DDX41 function is due to the formation process but not resolution of the R-loop itself.

### DDX41 promotes transcriptional elongation by facilitating cooperation between the spliceosome and transcriptional elongation complex

**Fig. 7:**
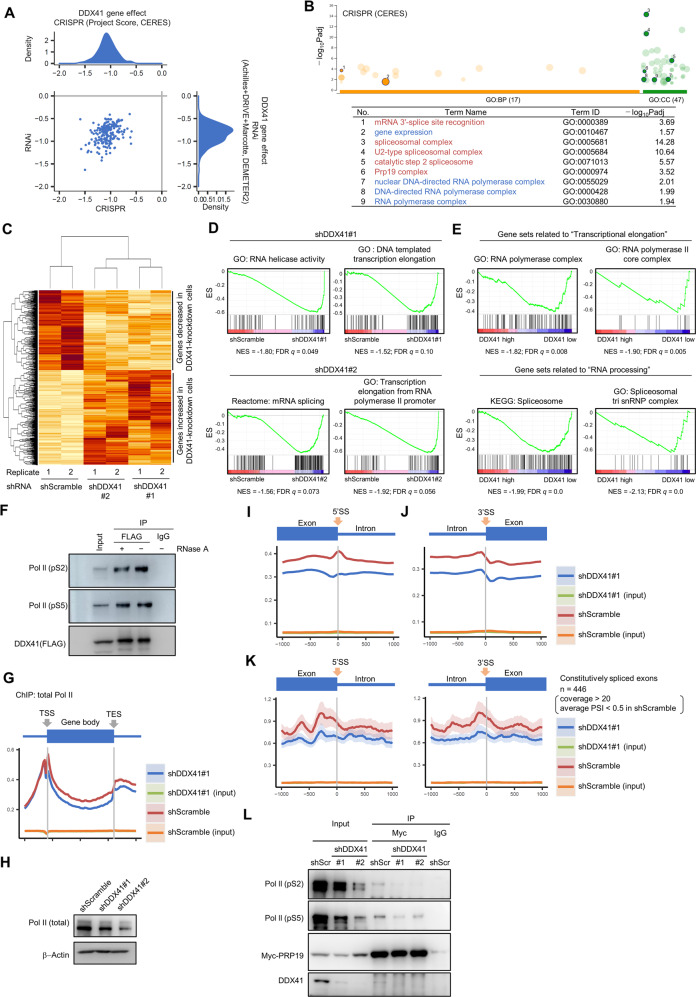
Changes in gene expression and distribution of Pol II by DDX41 knockdown. **A** Dependence of cell lines on DDX41. We used DepMap portal (https://depmap.org/portal/). For density distributions for CRISPR and RNAi data, smaller scores indicate that DDX41 is essential for cell line survival; −1 was comparable to the median of all pan-essential genes. **B** Genes co-dependent with DDX41 include those related to RNA splicing and Pol II-mediated transcription. Top co-dependent genes with DDX41 (with *q* values <0.05) identified in CRIPR screening were subjected to GO analysis; results were visualized via g:Profiler (upper). Representative GO terms related to RNA splicing (red) and Pol II-mediated transcription (blue) were numbered (lower). Table S1 gives the complete list. **C** Gene expression changes by DDX41 knockdown. A hierarchical clustering of 1341 genes that showed expression changes with *p* < 0.05 in common in shDDX41#1- and shDDX41#2-expressing DDX41-knockdown cells compared with shScramble-expressing control cells was visualized. **D** Representative gene sets associated with RNA splicing and transcriptional elongation negatively enriched in shDDX41#1- and shDDX41#2-expressing cells. ES, enrichment score; NES, nominal enrichment score. **E** Representative gene sets associated with transcriptional elongation and RNA processing negatively enriched in *DDX41* low-expressing AML cases. The 451 AML cases presented in the article by Tyner et al. [48] were divided into three groups according to the expression level of *DDX41*, and the transcriptome differences between groups with *DDX41* expression levels below mean −SD (DDX41 low) and above mean +SD (DDX41 high) were examined. **F** Direct interaction of DDX41 with Pol II in HEK293FT cells. Protein extracts from cells expressing FLAG-DDX41 were immunoprecipitated with anti-FLAG antibody or control IgG and then probed with anti-FLAG and anti-Pol II (pS2 and pS5) antibodies. **G** Average distribution of total Pol II from transcription start site (TSS) to transcription end site (TES) of all RefSeq transcripts visualized with Ngsplot. **H** Changes in Pol II expression in DDX41-knockdown HEK293FT cells. **I**, **J** Average distribution of Pol II around exon/intron boundaries. Distribution of Pol II around 5′SS (**I**) and 3′SS (**J**) of genes expressing above the median in control cells are shown. **K** Average distribution of Pol II around exon/intron boundaries of constitutively spliced exons. Exons that met requirements of coverage >20 and average PSI < 0.5 were selected, and average distributions of Pol II up to 1000 bases upstream and downstream of the 5′SS (left panel) and 3′SS (right panel) of exons were visualized with Ngsplot. **L** Reduced interaction of PRP19 with pS2- and pS5-modified Pol II. Protein extracts from DDX41-knockdown HEK293FT expressing Myc-tagged PRP19 were immunoprecipitated with anti-Myc antibody or control IgG. The samples were probed with anti-Pol II (pS2 and pS5), anti-Myc and anti-DDX41 antibodies.

To investigate the mechanism by which DDX41 suppression caused DNA replication stress with R-loop accumulation, we analyzed the Cancer Dependency Map (DepMap) dataset [43]. In support of our observations (Fig. 3B, C), the dataset showed that knockdown and knockout of DDX41 both broadly affected cell viability (gene effect scores for CRISPR and RNAi were −1.07 ± 0.18 and −0.84 ± 0.21, respectively) (Fig. 7A), which strongly suggests that DDX41 is essential for cell survival. GO analysis of 100 and 47 genes that co-depended with DDX41 in knockdown and knockout screens, respectively, showed that GO terms related to RNA Pol II-mediated transcription and RNA splicing, including catalytic step 2 spliceosome and splicing C complex, were significantly enriched (Fig. 7B, Supplementary Fig. S7A and Supplementary Table S2). These results suggested that DDX41 maintains cell survival through regulation of RNA splicing and Pol II-mediated transcription. Importantly, RNA splicing is tightly coupled with transcriptional elongation [44–46]. Indeed, we found an interaction of DDX41 with NTC components that were also involved in transcriptional elongation (Fig. 2C) [26].

This DepMap data may reflect our data obtained from transcriptome analyses of DDX41-knockdown cells (Fig. 7C). Gene set enrichment analysis (GSEA) [47] showed that gene sets related to transcriptional elongation, RNA helicase activity, and mRNA splicing were enriched among the genes down-regulated in DDX41-knockdown cells (Fig. 7D and Supplementary Fig. S7B). Comparable results were obtained via analyses of published datasets of 451 AML cases (Fig. 7E) [48]. In addition, with the same clinical dataset, we confirmed that many genes related to catalytic step 2 spliceosome and Pol II-mediated transcriptional elongation were interdependent with DDX41 expression (Supplementary Fig. S7C). Thus, reduced expression of the genes related to these processes in cells with lower DDX41 expression was likely due to negative feedback regulation.

To investigate the relationship between DDX41-mediated RNA splicing and transcriptional elongation, we performed IP-Western blotting. We found that DDX41 interacted with Pol II phosphorylated at S2 and S5 (pS2 and pS5) in the C-terminal domain (CTD) and that RNA did not mediate the interaction (Fig. 7F), raising a possibility that DDX41 coordinates RNA splicing and transcriptional elongation.

We then performed fractionation-assisted native chromatin IP and sequencing analysis (ChIP-seq) [49] with an antibody against CTD of Pol II (with only shDDX41#1-expressing K562 cells because few shDDX41#2-expressing cells were available). ChIP-seq data showed that the average distribution of Pol II on the gene body was reduced in DDX41-knockdown cells (Fig. 7G), which may be partially due to reduced Pol II expression in DDX41-knockdown cells (Fig. 7H). We analyzed Pol II occupancy around 5ʹSS and 3ʹSS; interestingly, Pol II had a peak at 5ʹSS in the control cells, which disappeared in DDX41-knockdown cells (Fig. 7I). For 3ʹSS, Pol II signals decreased after exon-intron junctions, but the control and DDX41-knockdown cells showed a comparable shift (Fig. 7J), which confirmed the significance of changes in Pol II occupancy at 5ʹSS. Loss of the 5ʹSS peak in DDX41-knockdown cells did not depend on the difference in gene expression levels between DDX41-knockdown and the control cells, because a similar pattern at 5ʹSS was observed even when we performed the same analysis for the exons of genes expressing above the median in DDX41-knockdown cells (Supplementary Fig. S7D). These observations, along with our CLIP-seq data (Fig. 1A) demonstrating selective binding of DDX41 to 5ʹSS, support a unique role for DDX41 around 5ʹSS.

We next studied whether observed signal accumulation of Pol II at 5ʹSS was related to RNA splicing. We selected constitutively skipped exons in adequately transcribed genes in control cells for analysis (Supplementary Fig. S7E). We found no clear peaks at 5ʹSS and 3ʹSS on such skipped exons (Fig. 7K), although a signal fluctuation occurred because of the small number of exons included in analysis. These observations indicated that Pol II globally paused at 5ʹSS in association with RNA splicing similar to previous report [50], but this phenomenon no longer took place when DDX41 expression was decreased, even though RNA splicing changes occurred only in a subset of exons (Fig. 1). This result may be attributed to the reduced interaction of PRP19 with pS2 and pS5 Pol II (Fig. 7L), especially with pS2 Pol II, which was supported by findings from other groups that RNA splicing depends on the interaction of PRP19 to Pol II CTD [51–54]. All our data suggest that DDX41 coordinates RNA splicing and transcriptional elongation at 5ʹSS, thereby inhibiting aberrant R-loop accumulation and subsequent DNA replication stress.

### DDX41 inhibition causes R-loop accumulation and DNA damage in primary hematopoietic cells

**Fig. 8:**
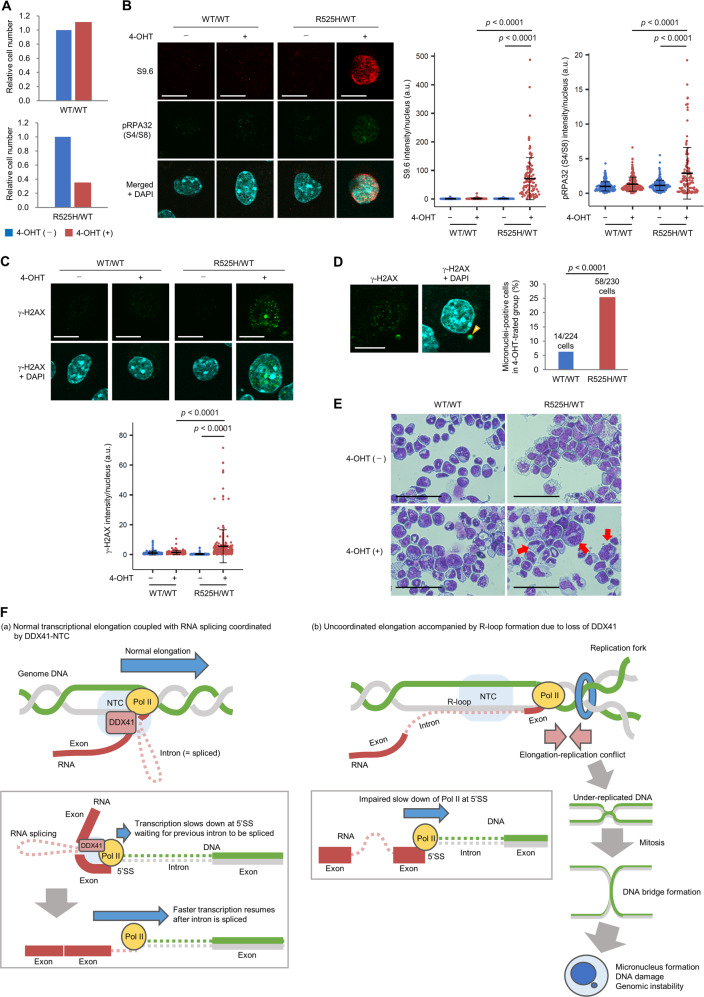
R-loop accumulation and DNA damage by introduction of R525H mutation in primary hematopoietic progenitor cells in mice. **A** Reduced proliferation of immature bone marrow cells expressing R525H mutation. Lin^−^/c-Kit^+^ bone marrow cells isolated from 9-week-old *Ddx41*^R525H/WT^ or *Ddx41*^WT/WT^ mice were cultured ex vivo in the presence of 25 ng/ml human thrombopoietin (TPO), 50 ng/ml mouse stem cell factor (SCF), 50 ng/ml mouse FLT3-L, and 25 mg/ml mouse interleukin-6 (IL-6) for 72 h, and then 4-OHT at 200 nM (final concentration) was added to culture medium for 48 h. Relative cell numbers compared with control cell numbers cultured in the presence or absence of 4-OHT are shown. **B**, **C** Increased R-loop formation and DNA damage in immature bone marrow cells expressing *Ddx41* R525H mutation. Cells cultured as in **A** were stained with S9.6 and anti-phospho-RPA32 (S4/S8) antibodies (**B**) or anti-γ-H2AX antibody (**C**) with nuclear DAPI counterstaining. Scale bars: 10 μm. (Right) Quantitative signal levels. *n* = 246, 217, 226 and 128 (**B**), and 238, 151, 190, and 290 (**C**) for WT/WT/4-OHT^−^, WT/WT/4-OHT^+^, R525H/WT/4-OHT^−^, and R525H/WT/4-OHT^+^, respectively; two-tailed Welch’s *t* test. **D** Micronucleus formation in immature bone marrow cells expressing *Ddx41* R525H mutation. Cells cultured as in **A** were stained with DAPI and anti-γ-H2AX antibody. (Left) Representative images of *Ddx41*^R525H/WT^ cell with γ-H2AX-positive micronucleus (arrowhead). Scale bar: 10 μm. (Right) Percent micronuclei-positive cells. Chi-square analysis. **E** Morphology of hematopoietic progenitor cells cultured ex vivo. Cells cultured as in **A** were stained with Giemsa and observed with light microscopy at ×400. Red arrows indicate cells with severely abnormal nuclear morphology. Scale bars: 50 μm. **F** Schematic illustration of how DDX41 deficiency causes impaired hematopoiesis and leukemogenesis. (a) Normal condition: DDX41, together with NTC, coordinates RNA splicing and transcriptional elongation. The Pol II complex transiently slows at 5ʹSS by interacting with DDX41, where it waits until the intron is spliced. (b) DDX41 deficiency: Pol II complex ignores unfinished RNA splicing and does not slow at 5ʹSS, which leads to increased opportunities for R-loop formation and transcription-replication conflicts. This results in increased under-replicated DNA at end of replication and DNA bridge formation during mitosis that result in genomic instability.

We developed *Ddx41*^R525H^ conditional knock-in (cKI) mice that express a Ddx41 mutant corresponding to human DDX41 R525H in a tamoxifen-inducible manner (Supplementary Fig. S8A, B) and examined its phenotypes of immature hematopoietic cells cultured ex vivo. Because our previous reports showed that R525H mutation reduced ATPase activity of DDX41 [8], we expected that induction of this mutant would reduce Ddx41 function. Lineage marker-negative/c-Kit-positive (Lin^−^/c-Kit^+^) immature bone marrow cells isolated from heterozygous (*Ddx41*^R525H/WT^) and WT (*Ddx41*^WT/WT^) mice were cultured in the presence of cytokines to support stem/progenitor cell growth, followed by induction of R525H mutation in *Ddx41*^R525H/WT^ cells by adding (Z)-4-hydroxytamoxifen (4-OHT). Induction of the mutant was confirmed by direct sequencing (Supplementary Fig. S8C). As in our previous study [8], induction of R525H mutation inhibited cell proliferation (Fig. 8A). Moreover, these cells showed increased R-loop formation, phosphorylation of replication protein A 32 (RPA32) at serine 4 and 8 residues—a marker of fork collapse [55]—and γ-H2AX signals (Fig. 8B, C), which confirmed that increased R-loop and DNA damage from DDX41 inhibition observed in cancer cell lines also occurred in primary immature hematopoietic cells. *Ddx41*^R525H/WT^ cells further showed increased micronucleus formation and abnormal nuclear morphology (Fig. 8D, E), which suggested involvement of loss of Ddx41 function in genomic instability. Finally, we observed R-loops in cultured hematopoietic cells from *Ddx41* heterozygous (*Ddx41*^WT/KO^) mice. Increased R-loop formation was observed in *Ddx41*^WT/KO^ cells, which was further enhanced by the induction of R525H mutation (*Ddx41*^R525H/KO^) (Supplementary Fig. S8D). These observations suggest that a reduction of DDX41 expression level causes certain dose-dependent disturbance and that R525H mutation is functionally hypomorphic.

In conclusion, our study identified a process by which loss of DDX41 expression or its helicase activity caused spliceosomal dysfunction and impaired transcriptional elongation, thus leading to DNA replication stress that resulted in genomic instability. Our data suggest a model in which DDX41 regulates Pol II pausing at the 5ʹSS with the NTC, and Pol II waits there for RNA splicing to complete, but if DDX41 is deficient, transcriptional elongation machinery may proceed without slowing down at 5ʹSS (Fig. 8F) or may terminate elongation and dissociate from chromatin. Although DNA replication defects in the absence of DDX41 are not necessarily severe, persistence and gradual accumulation of minor replication stress beyond mitosis would cause hematopoiesis failure that is often observed in bone marrow of patients with *DDX41* mutation.

## Discussion

Mutation or aberrant expression of genes encoding an RNA helicase occurs in various malignancies [56–58], which suggests that abnormal RNA recombination is closely associated with tumorigenesis. In hematopoietic malignancies, thus far, only *DDX41*, encoding a DEAD-box-type RNA helicase, and *DHX15* and *DHX34*, encoding DEAH-box-type helicases, were reproducibly mutated in AML/MDS [31, 59, 60]. Characterization of these gene products would provide an excellent model for clarifying biological roles of RNA helicases and its involvement in leukemogenesis.

Here, we discovered that inhibition of DDX41 led to DNA replication stress with increased R-loop formation. Although the degree of the stress was modest, cells underwent mitosis with under-replicated DNA, resulting in genomic instability and growth inhibition. Hematopoietic stem cells (HSCs) have relatively longer cell cycles and divide at a low rate, once every 40 weeks [61]. Therefore, most HSCs remain quiescent in a steady state and have fewer opportunities to enter S phase. However, HSCs become more likely to enter the cell cycle when they are exposed to inflammatory environments or when they are aged. Importantly, cycling aged HSCs in mice have higher levels of replication stress associated with cell cycle defects; such replication stress persists even after the cells re-establish quiescence [62]. Therefore, in the presence of *DDX41* mutations, even weak replication stress can accumulate in aged HSCs. In addition, considering the sequential acquisition of *DDX41* mutations observed in myeloid malignancies, hematopoietic cells with a heterozygous germline *DDX41* variant would have to wait until they acquire somatic mutations in other alleles to develop a myeloid malignancy, because DNA replication stress acquired in cells with a heterozygous variant may be limited even in aged HSCs. This was also suggested from the phenotypes observed in *Ddx41* genetically modified mice [7]. These ideas may explain why carriers of germline *DDX41* variants generally develop myeloid malignancy at older ages.

We also showed that DNA damage caused by loss of DDX41 function was dependent on transcription-related R-loop accumulation. Although impaired RNA splicing can lead to R-loop formation [63], its process remains poorly understood. Accumulating evidences implicate the interdependence of RNA splicing and transcriptional elongation [64]. Specifically, a recent study demonstrated that the upstream 5’SS remained associated to the transcription machinery during intron synthesis [50], consistent with our observation. Furthermore, Pol II paused at 5ʹSS resumed elongation after completion of RNA splicing [65]. Interestingly, our ChIP-seq analysis with antibody against Pol II showed that Pol II likely ignores pausing at 5ʹSS when not enough DDX41 is available. Taken together, we suggest that Pol II continues aberrant elongation without waiting for RNA splicing to finish, or alternatively, terminates elongation at 5ʹSS when RNA splicing is delayed by DDX41 deficiency. This model might be linked to a new perspective that the main obstacle to replication fork progression is the elongating Pol II engaged in R-loop [66].

DDX41 depletion induced mild splicing changes without any specific pattern; one possible explanation for this is that DDX41 regulates efficient splicing in the late step rather than decision of SS. However, further sequencing analysis of clinical specimens with *DDX41* mutation is clearly needed for its confirmation, given that *INTS3* intron retention observed in DDX41-knockdown cells was reported to be highly exclusive to *SRSF2* mutation [67]. Since only a subset of R-loops can be hotspots for DNA damage [68], regions and types of splicing defect may determine the impact on genomic stability. We conclude that the vulnerability in DNA replication that partially remains in daughter cells would be essential to explain the unique phenotype of *DDX41*-related myeloid malignancies.

## Materials and methods

### Cell lines

K562 and THP-1 cells were cultured in RPMI-1640 (Sigma-Aldrich) with 10% heat-inactivated fetal bovine serum. HEK293FT, HEK293T, HeLa, U2OS and ARPE-19 cells were cultured in Dulbecco’s Modified Eagle Medium (Sigma-Aldrich) with 10% heat-inactivated fetal bovine serum. All cells were maintained in a 5% CO_2_ incubator at 37 °C.

### DDX41 inhibitors

DDX41inh-1 and DDX41inh-2 were gifts from Axcelead Drug Discovery Partners Inc.

### Cell cycle synchronization

HeLa cells were synchronized to the late G2 phase or G1/S boundary by double thymidine block with or without CDK1 inhibitor, respectively (see Supplementary Methods).

### Flow cytometry

Cell cycle distribution, incorporation of BrdU or IdU, apoptosis and γ-H2AX expression were analyzed via flow cytometry (see Supplementary Methods).

### CLIP-seq and ChIP-seq analyses

CLIP-seq was performed according to a previously reported method (Supplementary Fig. S1A) [20, 21]. ChIP-seq was performed by applying the fractionation-assisted native ChIP method (49). Detailed procedures are described in Supplementary Methods.

### Mice

*Ddx41*^R525H^ cKI mice were crossed with C57BL/6-Gt(ROSA)26Sor^tm1(cre/Esr1)Arte^ (ERT2Cre) mice (Model6466, Taconic Biosciences) to allow tamoxifen-inducible excision of floxed regions. *Ddx41* heterozygous knockout mice (C57BL/6N Ddx41^tm1a^) were purchased from the UC Davis KOMP Repository (MMRRC Stock #047340-UCD). All mice were kept according to guidelines of the Institute of Laboratory Animal Science, Hiroshima University. The Animal Care Committee at the Japanese Foundation for Cancer Research approved all murine studies. Detailed procedures for generating *Ddx41*^R525H^ cKI mice and experimental procedures with the mice are described in Supplementary Methods.

### Statistical analysis

Statistical analysis was performed with R software (version 4.1.2). All statistical details of experiments are given in Supplementary Methods and corresponding figure legends, with *p* values ≤0.01 being considered statistically significant unless otherwise indicated. Error bars in figures indicate standard deviation (SD).

Additional methods are available in the Supplementary Information.

## Supplementary information

Supplementary information

Supplementary Movie 1

Supplementary Movie 2

Supplementary Movie 3

Supplementary Table S1

Supplementary Table S2

## Data Availability

RNA-seq, ChIP-seq, and CLIP-seq data are available in the sequence read archive (SRA) database (https://ddbj.nig.ac.jp/search) (accession ID for CLIP-seq: DRA011992, for RNA-seq: DRA014267, and for ChIP-seq: DRA014255). The datasets generated during the study are available from the corresponding author upon reasonable request.

## References

[1] Churpek JE, Smith-Simmer K. *DDX41*-Associated Familial Myelodysplastic Syndrome and Acute Myeloid Leukemia. In: Adam MP, Ardinger HH, Pagon RA, Wallace SE, Bean LJH, Gripp KW, et al. editors. University of Washington, Seattle: GeneReviews; 2021.

[2] Li P, White T, Xie W, Cui W, Peker D, Zeng G (2021). AML with germline DDX41 variants is a clinicopathologically distinct entity with an indolent clinical course and favorable outcome. Leukemia..

[3] Sébert M, Passet M, Raimbault A, Rahmé R, Raffoux E, Sicre de Fontbrune F (2019). Germline DDX41 mutations define a significant entity within adult MDS/AML patients. Blood..

[4] Yang F, Long N, Anekpuritanang T, Bottomly D, Savage JC, Lee T (2022). Identification and prioritization of myeloid malignancy germline variants in a large cohort of adult patients with AML. Blood..

[5] Mosler T, Conte F, Longo GMC, Mikicic I, Kreim N, Möckel MM (2021). R-loop proximity proteomics identifies a role of DDX41 in transcription-associated genomic instability. Nat Commun.

[6] Weinreb JT, Ghazale N, Pradhan K, Gupta V, Potts KS, Tricomi B (2021). Excessive R-loops trigger an inflammatory cascade leading to increased HSPC production. Dev Cell.

[7] Chlon TM, Stepanchick E, Hershberger CE, Daniels NJ, Hueneman KM, Kuenzi Davis A (2021). Germline DDX41 mutations cause ineffective hematopoiesis and myelodysplasia. Cell Stem Cell.

[8] Kadono M, Kanai A, Nagamachi A, Shinriki S, Kawata J, Iwato K (2016). Biological implications of somatic DDX41 p.R525H mutation in acute myeloid leukemia. Exp Hematol.

[9] Wahl MC, Will CL, Lührmann R (2009). The spliceosome: design principles of a dynamic RNP machine. Cell..

[10] Cvitkovic I, Jurica MS (2013). Spliceosome database: a tool for tracking components of the spliceosome. Nucleic Acids Res.

[11] Schmidt C, Grønborg M, Deckert J, Bessonov S, Conrad T, Lührmann R (2014). Mass spectrometry-based relative quantification of proteins in precatalytic and catalytically active spliceosomes by metabolic labeling (SILAC), chemical labeling (iTRAQ), and label-free spectral count. RNA..

[12] Bessonov S, Anokhina M, Krasauskas A, Golas MM, Sander B, Will CL (2010). Characterization of purified human Bact spliceosomal complexes reveals compositional and morphological changes during spliceosome activation and first step catalysis. RNA..

[13] Ma J, Mahmud N, Bosland MC, Ross SR (2022). DDX41 is needed for pre- and postnatal hematopoietic stem cell differentiation in mice. Stem Cell Rep.

[14] Alkhateeb HB, Nanaa A, Viswanatha D, Foran JM, Badar T, Sproat L (2022). Genetic features and clinical outcomes of patients with isolated and comutated DDX41-mutated myeloid neoplasms. Blood Adv.

[15] Duployez N, Largeaud L, Duchmann M, Kim R, Rieunier J, Lambert J (2022). Prognostic impact of DDX41 germline mutations in intensively treated acute myeloid leukemia patients: an ALFA-FILO study. Blood..

[16] Choi EJ, Cho YU, Hur EH, Jang S, Kim N, Park HS (2022). Unique ethnic features of DDX41 mutations in patients with idiopathic cytopenia of undetermined significance, myelodysplastic syndrome, or acute myeloid leukemia. Haematologica..

[17] Quesada AE, Routbort MJ, DiNardo CD, Bueso-Ramos CE, Kanagal-Shamanna R, Khoury JD (2019). DDX41 mutations in myeloid neoplasms are associated with male gender, TP53 mutations and high-risk disease. Am J Hematol.

[18] Badar T, Chlon T. Germline and somatic defects in DDX41 and its Impact on myeloid neoplasms. Curr Hematol Malig Rep. 2022;17:113–20. 10.1007/s11899-022-00667-3 PMC10324161 35781188

[19] Li P, Brown S, Williams M, White TA, Xie W, Cui W (2022). The genetic landscape of germline DDX41 variants predisposing to myeloid neoplasms. Blood..

[20] Van Nostrand EL, Nguyen TB, Gelboin-Burkhart C, Wang R, Blue SM, Pratt GA (2017). Robust, cost-effective profiling of RNA binding protein targets with single-end enhanced crosslinking and immunoprecipitation (seCLIP). Methods Mol Biol.

[21] Van Nostrand EL, Pratt GA, Shishkin AA, Gelboin-Burkhart C, Fang MY, Sundararaman B (2016). Robust transcriptome-wide discovery of RNA-binding protein binding sites with enhanced CLIP (eCLIP). Nat Methods.

[22] Shen S, Park JW, Lu ZX, Lin L, Henry MD, Wu YN (2014). rMATS: robust and flexible detection of differential alternative splicing from replicate RNA-Seq data. Proc Natl Acad Sci USA.

[23] Rahman MA, Lin KT, Bradley RK, Abdel-Wahab O, Krainer AR (2020). Recurrent SRSF2 mutations in MDS affect both splicing and NMD. Genes Dev.

[24] de Almeida RA, O’Keefe RT (2015). The NineTeen Complex (NTC) and NTC-associated proteins as targets for spliceosomal ATPase action during pre-mRNA splicing. RNA Biol.

[25] Chanarat S, Sträßer K (2013). Splicing and beyond: the many faces of the Prp19 complex. Biochim Biophys Acta.

[26] Chanarat S, Seizl M, Strässer K (2011). The Prp19 complex is a novel transcription elongation factor required for TREX occupancy at transcribed genes. Genes Dev.

[27] Busetto V, Barbosa I, Basquin J, Marquenet É, Hocq R, Hennion M (2020). Structural and functional insights into CWC27/CWC22 heterodimer linking the exon junction complex to spliceosomes. Nucleic Acids Res.

[28] Fica SM, Oubridge C, Wilkinson ME, Newman AJ, Nagai K (2019). A human postcatalytic spliceosome structure reveals essential roles of metazoan factors for exon ligation. Science..

[29] Tsukamoto T, Gearhart MD, Kim S, Mekonnen G, Spike CA, Greenstein D (2020). Insights into the involvement of spliceosomal mutations in myelodysplastic disorders from analysis of SACY-1/DDX41 in caenorhabditis elegans. Genetics..

[30] Weinreb JT, Gupta V, Sharvit E, Weil R, Bowman TV (2021). Ddx41 inhibition of DNA damage signaling permits erythroid progenitor expansion in zebrafish. Haematologica..

[31] Polprasert C, Schulze I, Sekeres MA, Makishima H, Przychodzen B, Hosono N (2015). Inherited and Somatic Defects in DDX41 in Myeloid Neoplasms. Cancer Cell.

[32] Paulsen RD, Soni DV, Wollman R, Hahn AT, Yee MC, Guan A (2009). A genome-wide siRNA screen reveals diverse cellular processes and pathways that mediate genome stability. Mol Cell.

[33] Yoneyama-Hirozane M, Kondo M, Matsumoto SI, Morikawa-Oki A, Morishita D, Nakanishi A (2017). High-Throughput Screening to Identify Inhibitors of DEAD Box Helicase DDX41. SLAS Discov.

[34] Sloan KE, Bohnsack MT (2018). Unravelling the mechanisms of RNA helicase regulation. Trends Biochem Sci.

[35] Wilhelm T, Olziersky AM, Harry D, De Sousa F, Vassal H, Eskat A (2019). Mild replication stress causes chromosome mis-segregation via premature centriole disengagement. Nat Commun.

[36] Böhly N, Kistner M, Bastians H (2019). Mild replication stress causes aneuploidy by deregulating microtubule dynamics in mitosis. Cell Cycle.

[37] Siri SO, Martino J, Gottifredi V (2021). Structural chromosome instability: types, origins, consequences, and therapeutic opportunities. Cancers..

[38] Crossley MP, Bocek M, Cimprich KA (2019). R-loops as cellular regulators and genomic threats. Mol Cell.

[39] Bou-Nader C, Bothra A, Garboczi DN, Leppla SH, Zhang J (2022). Structural basis of R-loop recognition by the S9.6 monoclonal antibody. Nat Commun.

[40] Chen L, Chen JY, Zhang X, Gu Y, Xiao R, Shao C (2017). R-ChIP using inactive RNase H reveals dynamic coupling of R-loops with transcriptional pausing at gene promoters. Mol Cell.

[41] Cristini A, Groh M, Kristiansen MS, Gromak N (2018). RNA/DNA hybrid interactome identifies DXH9 as a molecular player in transcriptional termination and R-loop-associated DNA damage. Cell Rep.

[42] Wang IX, Grunseich C, Fox J, Burdick J, Zhu Z, Ravazian N (2018). Human proteins that interact with RNA/DNA hybrids. Genome Res.

[43] Tsherniak A, Vazquez F, Montgomery PG, Weir BA, Kryukov G, Cowley GS (2017). Defining a cancer dependency map. Cell..

[44] Saldi T, Cortazar MA, Sheridan RM, Bentley DL (2016). Coupling of RNA polymerase II transcription elongation with pre-mRNA splicing. J Mol Biol.

[45] Custódio N, Carmo-Fonseca M (2016). Co-transcriptional splicing and the CTD code. Crit Rev Biochem Mol Biol.

[46] Caizzi L, Monteiro-Martins S, Schwalb B, Lysakovskaia K, Schmitzova J, Sawicka A (2021). Efficient RNA polymerase II pause release requires U2 snRNP function. Mol Cell.

[47] Subramanian A, Tamayo P, Mootha VK, Mukherjee S, Ebert BL, Gillette MA (2005). Gene set enrichment analysis: a knowledge-based approach for interpreting genome-wide expression profiles. Proc Natl Acad Sci USA.

[48] Tyner JW, Tognon CE, Bottomly D, Wilmot B, Kurtz SE, Savage SL (2018). Functional genomic landscape of acute myeloid leukaemia. Nature..

[49] Miyamoto R, Yokoyama A (2021). Protocol for fractionation-assisted native ChIP (fanChIP) to capture protein-protein/DNA interactions on chromatin. STAR Protoc.

[50] Leader Y, Lev Maor G, Sorek M, Shayevitch R, Hussein M, Hameiri O (2021). The upstream 5’ splice site remains associated to the transcription machinery during intron synthesis. Nat Commun.

[51] David CJ, Boyne AR, Millhouse SR, Manley JL (2011). The RNA polymerase II C-terminal domain promotes splicing activation through recruitment of a U2AF65-Prp19 complex. Genes Dev.

[52] Hsin JP, Manley JL (2012). The RNA polymerase II CTD coordinates transcription and RNA processing. Genes Dev.

[53] Hirose Y, Tacke R, Manley JL (1999). Phosphorylated RNA polymerase II stimulates pre-mRNA splicing. Genes Dev.

[54] Millhouse S, Manley JL (2005). The C-terminal domain of RNA polymerase II functions as a phosphorylation-dependent splicing activator in a heterologous protein. Mol Cell Biol.

[55] Ashley AK, Shrivastav M, Nie J, Amerin C, Troksa K, Glanzer JG (2014). DNA-PK phosphorylation of RPA32 Ser4/Ser8 regulates replication stress checkpoint activation, fork restart, homologous recombination and mitotic catastrophe. DNA Repair.

[56] Cargill M, Venkataraman R, Lee S (2021). DEAD-Box RNA helicases and genome stability. Genes..

[57] He Y, Zhang D, Yang Y, Wang X, Zhao X, Zhang P (2018). A double-edged function of DDX3, as an oncogene or tumor suppressor, in cancer progression (Review). Oncol Rep.

[58] Li F, Fountzilas C, Puzanov I, Attwood KM, Morrison C, Ling X (2021). Multiple functions of the DEAD-box RNA helicase, DDX5 (p68), make DDX5 a superior oncogenic biomarker and target for targeted cancer therapy. Am J Cancer Res.

[59] Faber ZJ, Chen X, Gedman AL, Boggs K, Cheng J, Ma J (2016). The genomic landscape of core-binding factor acute myeloid leukemias. Nat Genet.

[60] Rio-Machin A, Vulliamy T, Hug N, Walne A, Tawana K, Cardoso S (2020). The complex genetic landscape of familial MDS and AML reveals pathogenic germline variants. Nat Commun.

[61] Catlin SN, Busque L, Gale RE, Guttorp P, Abkowitz JL (2011). The replication rate of human hematopoietic stem cells in vivo. Blood..

[62] Flach J, Milyavsky M (2018). Replication stress in hematopoietic stem cells in mouse and man. Mutat Res.

[63] Chen L, Chen JY, Huang YJ, Gu Y, Qiu J, Qian H (2018). The augmented R-loop is a unifying mechanism for myelodysplastic syndromes induced by high-risk splicing factor mutations. Mol Cell.

[64] Muniz L, Nicolas E, Trouche D (2021). RNA polymerase II speed: a key player in controlling and adapting transcriptome composition. EMBO J.

[65] Nojima T, Gomes T, Grosso ARF, Kimura H, Dye MJ, Dhir S (2015). Mammalian NET-seq reveals genome-wide nascent transcription coupled to RNA processing. Cell..

[66] Zardoni L, Nardini E, Brambati A, Lucca C, Choudhary R, Loperfido F (2021). Elongating RNA polymerase II and RNA:DNA hybrids hinder fork progression and gene expression at sites of head-on replication-transcription collisions. Nucleic Acids Res.

[67] Yoshimi A, Lin KT, Wiseman DH, Rahman MA, Pastore A, Wang B (2019). Coordinated alterations in RNA splicing and epigenetic regulation drive leukaemogenesis. Nature..

[68] Costantino L, Koshland D (2018). Genome-wide map of R-loop-induced damage reveals how a subset of R-loops contributes to genomic instability. Mol Cell.

