# Supplementary material for: DDX41 coordinates RNA splicing and transcriptional elongation to prevent DNA replication stress in hematopoietic cells
Source: Leukemia. 2022 Oct 14;36(11):2605–20. doi: 10.1038/s41375-022-01708-9 (PMC9613458; doi:10.1038/s41375-022-01708-9)
Supplement: Supplementary file 1 — Supplementary information [file 41375_2022_1708_MOESM1_ESM.pdf]

Supplementary Information

DDX41 coordinates RNA splicing and transcriptional elongation to prevent DNA replication stress in hematopoietic cells

Shinriki et al.

This PDF file includes:

1. Supplementary Methods
2. Supplementary Figures 1-8
3. Legends for Supplementary Movies 1-3
4. Supplementary References

Supplementary Methods

Cell lines. HEK293FT was purchased from ThermoFisher Scientific. HEK293T and HeLa were purchased from American Type Culture Collection. K562, THP-1 and U2OS were gifts of Toshiya Inaba (Hiroshima University). ARPE-19 was a gift of Masayoshi Tasaki (Kumamoto University, Japan). K562 and THP-1 cells were cultured in RPMI-1640 (Sigma-Aldrich) with 10% heat-inactivated fetal bovine serum. HEK293FT, HEK293T, HeLa, U2OS and ARPE-19 cells were cultured in Dulbecco's Modified Eagle Medium (Sigma-Aldrich) with 10% heat-inactivated fetal bovine serum. All cells were maintained in a 5% CO_2 incubator at 37 °C.

Infection of shRNAs. shRNAs targeting DDX41 (shDDX41#1 and #2) and a negative control shRNA (shScramble) were inserted into pLKO.1-puro lentivirus vector. shRNA-pLKO.1-puro was co-transfected with psPAX2 and pMD2G into HEK293FT cells using Lipofectamine 2000 (Invitrogen). After 16 h of incubation, supernatant was replaced with fresh medium, and 48 h after medium replacement, supernatant was collected and filtered with a 0.45-μm filter. Virus in the supernatants was used to infect K562 and THP-1 cells, and cells were cultured in the presence of 1.5 μg/ml puromycin. Target sequences for DDX41 knockdown were as follows: 5′-GCCACTACCTTCATCAACAAA-

3′ for shDDX41#1, and 5′-GAGGAGATTGAGAACTATGTA-3′ for shDDX41#2. shScramble was a gift from Dr. David Sabatini (Addgene, #1864) [1].

Transfection with siRNAs. siRNAs targeting DDX41 (siDDX41#1, SASI_Hs01_00161700; siDDX41#2, SASI_Hs01_00161702) and a negative control siRNA (siControl, AM4611) were purchased from Sigma-Aldrich. Lipofectamine 2000 was used for transfection according to the manufacturer's instructions.

Nucleofection. K562 cells infected with shDDX41 and selected with puromycin were transfected with p3×FLAG-CMV14 plasmid encoding WT- or D210N-RNase H1 using Amaxa Cell Line Nucleofector Kit V (Lonza) with the Nucleofector IIb device (Lonza; program T-016) according to the manufacturer's instructions.

Cell growth assay. K562 cells and THP-1 cells were infected with lentivirus expressing shRNA for 48 h, and living cells after 48-h puromycin selection were seeded into a 96-well plate. HeLa cells were plated in a 96-well plate 24 h before siRNA transfection. Cell Counting Kit-8 reagent (Dojindo Molecular Technologies) was added to each well; absorbance at 450 nm was measured by using a microplate reader (Wako). The

proliferation level was normalized against the measurement made on the first day. When indicated, the number of living cells was counted by using trypan blue.

Cell cycle synchronization and drug treatment. To synchronize HeLa cells to the G1/S boundary, cells were treated with 2 mM thymidine (Nacalai Tesque) for 18 h. After releasing cells for 9 h, thymidine was added for another 15 h. To inhibit DDX41 or treat with APH in S phase, cells released from the second thymidine block were added with dimethyl sulfoxide (DMSO), 50 μM DDX41inh-2, or 10 nM APH. When indicated, IdU (Tokyo Chemical Industry) was added 20 min before cell harvest (see Fig. 4C). To assess accumulation of single-stranded DNA induced by DDX41 inhibition or APH treatment, BrdU (10 μM) was incorporated for 40 min 6 h after release from G1/S boundary in the presence of DMSO, DDX41inh-2, or APH.

To analyze effects of DDX41 inhibition during S phase on subsequent mitosis and cell cycle progression of daughter cells, medium containing DMSO or DDX41inh-2 was replaced with fresh medium 8 h after release from the second thymidine block, and mitotic cells were fixed for immunofluorescence analysis. IdU incorporation (20 min) of daughter cells were analyzed 28 h after release from the second thymidine block (Supplementary Fig. S5A).

For analysis of G2-M transition rate, cells were released from the second thymidine block in the presence of DMSO or DDX41inh-2 for 6 h, after which RO-3306 (10 μM; Tokyo Chemical Industry), a CDK1 inhibitor, was added to prevent cells from entering mitosis. Ten hours after release from the second thymidine block, cells were washed three times with medium, followed by replacement with fresh medium for 1 h to let cells enter mitosis (Fig. 4I). Alternatively, cell cycle distribution of daughter cells was analyzed 18 h after removal of DDX41inh-2 and RO-3306.

For analysis of DNA damage in daughter G1 cells, 15 h after release from the second thymidine block, cells were exposed again to 2 mM thymidine for 13 h to trap daughter cells in G1 phase or at the G1/S boundary (Supplementary Fig. 5B). The percent pHH3-positive mitotic cells was evaluated. G2 phase arrest was confirmed by enrichment of 4N cells positive for the G2 phase marker CENP-F (Supplementary Fig. S4G).

To determine the APH concentration to induce mild replication stress, asynchronized HeLa cells were treated for 16 h with 0, 1, 10, or 50 nM APH and then treated with 100 ng/ml nocodazole for 3 h before fixation to trap cells in mitosis.

Flow cytometry. For cell cycle analysis, cells were harvested and fixed in 70% ice-cold

ethanol overnight at −30 °C. After cells were washed with phosphate-buffered saline (PBS) containing 0.5% bovine serum albumin (BSA) (0.5% BSA/PBS), they were stained with propidium iodide (PI) (Dojindo Molecular Technologies) and concomitantly treated with 20 μg/ml RNase A for 20 min at room temperature (RT) in the dark. For staining with pHH3 and CENP-F, fixed cells were washed with 0.5% BSA/PBS, incubated with primary antibodies against pHH3 (1:300) (650801; BioLegend) and CENP-F (1:200) (NB500-101, Novus Biologicals), and probed with Alexa Fluor 488-conjugated goat anti-mouse antibody (1:200) (A11029, ThermoFisher Scientific) and DyLight 649-conjugated donkey anti-rabbit antibody (1:200) (406406; BioLegend), followed by staining with PI. To quantify pHH3- and γ-H2AX-positive cells at separate cell cycle stages, cells were fixed in 70% ice-cold ethanol overnight at 4 °C, permeabilized with 0.5% Triton X-100 (Sigma-Aldrich) for 10 min at RT, incubated with primary antibodies against pHH3 (1:300) and γ-H2AX (1:200) (A700-053; Bethyl Laboratories), probed with secondary antibodies, and stained with PI. All steps were followed by washing with 0.5% BSA/PBS.

For IdU and BrdU incorporation assays, cells were incubated with 10 μM BrdU (BioLegend) or 10 μM IdU for 30 min or 20 min, respectively, before fixation with 70% ice-cold ethanol, and then treated with 2N HCl for 30 min at 37 °C to denature DNA. After cells were washed, they were stained with mouse anti-BrdU antibody and PI.

For apoptosis analysis, 10 days after infection with lentivirus expressing shDDX41#1, shDDX41#2, or shScramble, K562 cells were harvested and washed twice with PBS. Equal cell numbers were resuspended in tubes, and cells were stained with Annexin V-APC and 7-AAD (BD Biosciences). Samples were analyzed with FACSVerse flow cytometry (BD Biosciences) and FlowJo software (Tree Star).

RNA-FISH. A 50-mer oligo(dT) was purchased from FASMAC. Tailing of digoxigenin (DIG) to oligo(dT) was performed with DIG Oligonucleotide Tailing Kit, 2nd generation (Roche), according to the manufacturer's instructions. In brief, oligo(dT) was incubated with DIG-dUTP, dATP, and terminal transferase at 37 °C for 15 min, followed by addition of 0.2 M EDTA (pH8.0) to stop the reaction, with storage at −30 °C until use. HeLa cells were seeded on glass slides and transfected with siRNA for 48 h. Cells were fixed with 4% paraformaldehyde (PFA) and then treated with proteinase K. The cells were fixed again with 4% PFA, followed by acetylation solution treatment. Cells were prehybridized with yeast tRNA containing prehybridization solution for 2 h at 55 °C and were then incubated with prepared oligo(dT) probe in hybridization solution overnight at 55 °C. After washing and blocking samples, an anti-DIG antibody was added, and samples were incubated for 1 h at RT and then washed with PBS. Alexa fluor 594-conjugated secondary

antibody was added and incubated for 1 h at RT in the dark. 4′,6-Diamidino-2-phenylindole (DAPI) was added and incubated for 10 min at RT. Cells were washed with PBS containing 0.2% Tween-20, and images were captured via confocal microscopy (LCV110, Olympus)

DNA fiber assay. Exponentially growing or synchronized HeLa cells were labeled with consecutive pulses of 5-chloro-2'-deoxyuridine (CldU) (25 μM) and IdU (250 μM), each for 20 min. Cells were then trypsinized and resuspended in cold PBS. Cell suspension samples of 2 μl (about 800 cells) were spotted onto Fine Frost glass slides (Matsunami Glass), air-dried for 5 min, and then lysed in 7 μl of buffer containing 200 mM Tris-HCl, pH 7.5; 50 mM EDTA; and 0.5% sodium dodecyl sulfate (SDS) for 2 min. Slides were tilted at an angle to allow DNA to run slowly down slides. Slides were air-dried before fixation in 3:1 methanol:acetic acid. DNA fiber spreads were denatured with 2.5 M HCl for 80 min before blocking in 5% BSA/PBS for 1 h. Slides were then incubated with rat anti-BrdU antibody (ab6326; Abcam) at 1:500 and mouse anti-BrdU antibody (347580; BD Biosciences) at 1:25 for 2 h at RT to detect CldU and IdU, respectively. Slides were washed three times with 0.05% Tween-20 and PBS (PBST), then with PBS for 5 min per wash, followed by incubation with Alexa Fluor 488-conjugated donkey anti-rat antibody

(A21208; ThermoFisher Scientific) and Alexa Fluor 647-conjugated goat anti-mouse antibody (A21235; ThermoFisher Scientific) for 1 h at RT in the dark. After slides were washed three times with PBST, then with PBS for 5 min per wash, they were mounted with Vectashield mounting medium (Vector Laboratories). Images were captured with the FV3000 confocal microscope (Olympus) and a 100× oil immersion objective. Track lengths of the IdU-labeled tracks in double-labeled replication forks were analyzed manually with ImageJ software. To convert from μm/min to kb/min, a commonly used conversion factor of 2.59 kb/μm was applied [2].

Immunoprecipitation and western blot analysis. Protein from each cell line was extracted by using NP40 buffer (150 mM NaCl, 50 mM Tris-HCl pH 8.0, 1% NP-40) with a protease inhibitor cocktail (Roche). Cell lysates were centrifuged at 10,000 × *g* for 5 min at 4 °C, and supernatants were collected. Proteins were denatured by boiling with SDS-containing sample buffer (NP40 lysis buffer containing 1% SDS, 0.5% β-mercaptoethanol and 12.5% glycerol). For immunoprecipitation, 20 μl of Protein G Dynabeads (ThermoFisher Scientific) conjugated with an antibody (4 μg) were mixed with cell extracts overnight at 4 °C. The beads were washed six times with NP40 buffer, and then re-suspended with 40 μl SDS-containing sample buffer, followed by

denaturation and elution by heating at 95 °C for 3 min. Where indicated, immunoprecipitated samples were treated with 20 μg/ml RNase A for 30 min at 37 °C during wash step to remove RNA. Protein samples were separated by SDS-polyacrylamide gel electrophoresis (PAGE) and transferred onto polyvinylidene fluoride membranes (BioRad). Membranes were blocked with 5% skim milk and 0.1% Tween-20 (Sigma-Aldrich) in Tris-buffered saline (TBS) (pH 7.4), after which they were incubated with primary antibodies overnight at 4 °C. After membranes were washed three times with TBS containing 0.1% Tween-20, they were incubated with a corresponding secondary antibody conjugated with horseradish peroxidase for 1 h at RT. Images were developed via ECL Plus Chemiluminescence kit (GE Healthcare). The following primary antibodies were used: DDX41 (1:1000) (ab182007; Abcam), γ-H2AX (1:1000) (A700-053; Bethyl Laboratories), Chk1-pS317 (1:1000) (12302, Cell Signaling Technology), Chk1, (1:1000) (sc-8408; Santa Cruz Biotechnology), Pol II CTD4H3 specific for pS5 (1:1000) (sc-47701; Santa Cruz Biotechnology), Pol II 3E10 specific for pS2 (1:1000) (04-1571; Millipore), Pol II 8WG16 specific for unphosphorylated CTD repeats (1:1000) (sc-56767; Santa Cruz Biotechnology), FLAG (1:2000) (F1804; Sigma-Aldrich), Myc (1:1000) (M047-3; MBL), and β-Actin (1:5000) (A5441; Sigma-Aldrich).

Purification of DDX41 interacting proteins. Subcellular fractions of HEK293T cells were obtained by cytoskelton (CSK) buffer extraction and micrococcal nuclease (MNase) treatment as previously described [3]. HEK293T cells cultured in a 10-cm dish were resuspended in 1 ml CSK buffer (100 mM NaCl, 10 mM PIPES [pH 6.8], 3 mM $MgCl_2$, 1 mM EGTA [pH 7.6], 0.3 M sucrose, 0.5% Triton X-100, 5 mM sodium butyrate, 0.5 mM DTT, and EDTA-free protease inhibitor cocktail [Roche]), incubated on ice for 5 min, and then centrifuged (400 × *g*, 4 °C, 4 min). The supernatant was removed, and the pellet was resuspended in 1 ml MNase buffer (50 mM Tris-HCl [pH 7.5], 4 mM $MgCl_2$, 1 mM $CaCl_2$, 0.3 M sucrose, 5 mM sodium butyrate, 0.5 mM DTT, and protease inhibitor cocktail). One unit of MNase (Sigma-Aldrich) was added to the suspension, and the mixture was incubated at 37 °C for 10 to 12 min to obtain mono-nucleosomes. The MNase reaction was stopped by adding EDTA (pH 8.0) at the final concentration of 20 mM. The reaction mixture was centrifuged (17,000 × *g*, 4 °C, 5 min) to separate the supernatant (nucleosome fraction) and the pellet. The nucleosome fractions were subjected to immunoprecipitation using anti-FLAG antibody (F1804), washed five times with MNase buffer containing 20 mM EDTA, and then eluted in elution buffer (1% SDS and 50 mM $NaHCO_3$). The eluted material was analyzed by mass spectrometry using an LTQ Orbitrap ELITE ETD mass spectrometer (ThermoFisher Scientific).

Immunofluorescence. Adherent cells were grown on sterile 18-mm glass coverslips, fixed in 4% PFA in PBS for 10 min at RT, washed once with PBS, permeabilized for 20 min at RT in 0.5% Triton X-100 with PBS, and washed twice with PBS. For staining of K562 cells, cells fixed with 4% PFA were attached to glass slides by using the Cytospin 4 Centrifuge (ThermoFisher Scientific) before permeabilization. All primary and secondary antibodies were diluted in 2% BSA/PBS. Slides were incubated with primary antibodies overnight at 4 °C, followed by incubation with secondary antibodies for 1 h at RT in the dark. Slides were then washed with PBS and incubated for 5 min with DAPI in PBS at RT to stain DNA. Slides were mounted with Vectashield mounting medium (Vector Laboratories) and sealed with clear nail polish. Slides were stored at −20 °C in the dark until being studied with confocal microscopy (FV3000), and images were analyzed with ImageJ software. The following primary antibodies were used: γ-H2AX (rabbit, A700-053, 1:200; mouse, BioLegend 613401, 1:200), S9.6 (mouse, MABE1095, Millipore, 1:200), Nucleolin (rabbit, NB100-1920, Novus Biologicals, 1:200), RPA32-phospho-S4/S8 (rabbit, A300-245A, Bethyl Laboratories, 1:200), BLM (mouse, sc-365753, Santa Cruz, 1:200), α-Tubulin (mouse, 627902, BioLegend, 1:200), γ-Tubulin (rabbit, 620901, BioLegend, 1:200), Centrin 2 (rat, 698601, BioLegend, 1:200), and DDDDK

(rabbit, PM020, MBL, 1:200). To stain DNA-RNA hybrids, cells were fixed in ice-cold methanol for 7 min at −20 °C. Fixed cells were blocked with 2% BSA/PBS and then processed as described above. To visualize single-stranded DNA, cells were harvested and fixed with 4% PFA and permeabilized as described above, followed by staining with mouse anti-BrdU antibody (347580; BD Biosciences, 1:300) under non-denaturing conditions [4, 5]. Secondary antibodies and dilutions were as follows: Alexa Fluor 488 goat anti-rabbit (A11034, ThermoFisher Scientific, 1:200), Alexa Fluor 488 goat anti-mouse (1:200), Alexa Fluor 488 donkey anti-rat (1:200), Alexa Fluor 568 goat anti-rabbit (A11036, ThermoFisher Scientific, 1:200), Alexa Fluor 546 goat anti-mouse (A11030, ThermoFisher Scientific, 1:200), DyLight 649 donkey anti-rabbit (1:200), and Alexa Fluor 647 goat anti-mouse (1:200).

Time lapse imaging. U2OS cells expressing histone-H2B fused with GFP were seeded on a glass-bottom dish and incubated in a CO_2 incubator equipped with an LCV110 microscope (Olympus). Images were captured every 10 min.

Analysis of gene expression dataset. A normalized expression dataset of 451 AML cases was obtained from a supplemental table in a recent report [6] and cBioPortal [7, 8]. We

divided the AML cases into three groups on the basis of DDX41 expression levels and examined differences in transcriptomes in groups with DDX41 expression levels below mean−SD and above mean+SD by using Gene Set Enrichment Analysis (GSEA).

We also analyzed the dataset from the DepMap database (https://depmap.org/portal/) [9]. The gene effect scores of DDX41 were derived from two screening experiments, DEMETER2 (RNAi) and CERES (CRISPR), and we analyzed the functional dependence on DDX41 across hundreds of human cancer cell lines. Negative effect scores represent slower growth, whereas positive scores show faster growth after knockdown or knockout of DDX41. Genes showing co-dependence with DDX41 ($q < 0.05$) were extracted from both screening datasets to form signature gene sets for estimating the core biological function of DDX41. Genes showing co-dependence with DDX41 were submitted to g:Profiler (https://biit.cs.ut.ee/gprofiler) [10] to estimate and visualize functional enrichment of the genes.

RNA-sequencing (RNA-seq) and RNA splicing analysis. Total RNA was extracted from K562 cells transduced with shDDX41#1, shDDX41#2, or shScramble or expressing SRSF2 P95R. mRNA was enriched by using oligo(dT) beads, followed by random fragmentation and cDNA synthesis with mRNA template and random hexamer primers.

After second-strand synthesis and adapter ligation, a double-stranded cDNA library was prepared by size selection and polymerase chain reaction enrichment. RNA-seq was performed with the HiSeq2500 sequencer (Illumina) with a 150-base pair, paired-end protocol. Sequenced reads were aligned to the human reference genome (hg38) by using STAR [11] and visualized via Integrative Genomics Viewer (IGV) [12]. Differential expression analysis was performed with edgeR [13], and rMATS [14] and maser (https://github.com/DiogoVeiga/maser) were used for differential RNA splicing analysis, and MEME [15] was used for motif identification. DAVID [16], GSEA [17], and Cytoscape [18] were used for GO analysis, GSEA, and visualization, respectively.

Crosslinking, immunoprecipitation and sequencing (CLIP-seq) analysis. CLIP-seq was performed according to a previously reported method (Supplementary Fig. S1A) [19, 20]. Briefly, 2.0×10^7 K562 cells expressing Myc-tagged WT or R525H mutant DDX41 were irradiated with 400 mJ/cm^2 UV light (wavelength: 254 nm) and were lysed, and RNA was fragmented by using RNase I. Crosslinked Myc-tagged DDX41-RNA complex in supernatants of lysate after centrifugation was then precipitated with protein G beads conjugated with 5 μg of anti-Myc antibody, followed by 3′ adaptor ligation to captured RNA on the beads. The protein-RNA complex was then subjected to PAGE and

transferred to a membrane. The 70- to 150-kDa region containing Myc-DDX41 with crosslinked RNA was excised from the membrane, and RNA was extracted after proteinase K treatment, after which RNA was reverse transcribed and libraries were prepared. Libraries were sequenced with the NovaSeq sequencer (Illumina). Prior to read mapping, the 3' adaptor sequences were trimmed from the sequenced reads using cutadapt [21]. The trimmed reads were aligned to the human genome (hg38) and rDNA (NR_145819.1, NR_023363.1) using STAR. UMI-tools [22] was used for PCR-duplicate removal. For the mapped reads, the types of genomic loci were analyzed using MultiQC [23].

Chromatin immunoprecipitation and sequencing (ChIP-seq) analysis. ChIP-seq analysis was performed by applying the fractionation-assisted native ChIP method [24]. Briefly, DDX41-knockdown K562 cells and shScramble-introduced K562 cells were lysed, centrifuged, and treated with MNase to purify the soluble chromatin fraction. Subsequently, anti-Pol II antibody (clone 8WG16, 05-952-I, Merck) was added to the chromatin fraction to perform IP, and bound DNA was extracted and processed for library preparation. Sequencing was performed by using the NovaSeq sequencer and the 100-base pair, single-end protocol. Sequenced reads were aligned to the human reference

genome (hg38) via Bowtie2 [25] and were visualized with IGV [12]. Ngsplot [26] was used for region-specific analysis of ChIP-seq reads and visualization.

Construction of a targeting vector and generation of *Ddx41*^{R525H} conditional knock-in (cKI) mice. *Ddx41*^{R525H} targeting vector was cloned by using an *Escherichia coli* recombination approach according to the manufacturer's protocol. A bacterial artificial chromosome (BAC) clone containing mouse *Ddx41* was purchased from BACPAC Resources Center of Children's Hospital Oakland Research Institute. *Ssp*I-*Eco*RI (4.0 kb) and *Sac*I-*Sac*I (5.0 kb) fragments of *Ddx41* were used as the 5′ and 3′ arms of the targeting vector. As Fig. S8A shows, a floxed *Ddx41* WT cDNA (downstream of exon 11) with SV40-polyA (PA), a mutant *Ddx41* cDNA (downstream of exon 11) carrying the R525H missense mutation (CGT > CAT in exon 15) with SV40-PA, and a Flpe recombinase target (FRT)-flanked neomycin resistance (Neo) gene were inserted between the two arms. A diphtheria toxin-A gene was attached to the 5′-end for negative selection. The integrity of the targeting vector was confirmed by polymerase chain reaction amplification and DNA sequencing of cassette junctions as well as by restriction enzyme digestion. KY1.1 ES cells (provided by Dr. Honda at Tokyo Women's Medical University) were electroporated with the targeting vector and subjected to G418

selection. Homologous recombination within ES clones was identified by Southern blotting analysis of *Bam*HI-digested genomic DNA and *Hin*dIII-digested genomic DNA with 5′ and 3′ probes (Supplementary Fig. S8B). Correctly targeted ES clones were injected into C57BL/6N mouse blastocysts, and chimeric males were mated with C57BL/6N females to transmit the targeted allele. FRT-flanked Neo was removed by crossing the heterozygotes with *CAG-FLPe* transgenic mice (RBRC01834, RIKEN BRC), which generated a floxed allele.

Established *Ddx41*^{R525H} cKI mice were crossed with C57BL/6-Gt(ROSA)26Sor$^{tm1(cre/Esr1)Arte}$ (ERT2Cre) mice (Model6466, Taconic Biosciences) to allow tamoxifen-inducible excision of floxed regions. To confirm the induction of the R525H mutation only by tamoxifen treatment, Sanger sequencing analysis of cDNA isolated from tamoxifen-treated Lin^{-} bone marrow cells from *Ddx41*^{R525H} cKI mice was performed by using the following sequencing primers: forward: 5′-GTACCATCTTCTCCTACTTCAAG-3′; reverse: 5′-CAGAACAGGTGGTACCTTCTG-3′ (Supplementary Fig. S8C). All mice were kept according to guidelines of the Institute of Laboratory Animal Science, Hiroshima University. The Animal Care Committee at the Japanese Foundation for Cancer Research approved all murine studies. All mice were housed in groups of three to five animals per cage and maintained on a 12-h light/dark

cycle at 20-25 °C. *Ddx41*^{R525H} cKI mice were deposited in the RIKEN BioResource Center (Tsukuba, Japan) (Record number: RBRC10077).

Cell culture of murine immature primary hematopoietic cells. Bone marrow cells were obtained from the femurs and tibias of the 9 weeks-old male mice, and red blood cells in the samples were lysed with BD Pharm Lyse (BD Biosciences). Cells were captured with biotin-conjugated anti-lineage antibody cocktail combined with anti-Biotin MicroBeads, and cells that were not captured in this process were used in the study. Lin^{-} immature bone marrow cells isolated from *Ddx41*$^{R525H/WT}$ or *Ddx41*$^{WT/WT}$ mice were cultured ex vivo in bone marrow culture medium containing Iscove's modified Dulbecco's medium (Sigma-Aldrich), 10% fetal bovine serum, 1% penicillin-streptomycin solution (Nacalai Tesque), HEPES, and 2-mercaptoethanol in the presence of 25 ng/ml human TPO, 50 ng/ml mouse SCF, 50 ng/ml mouse FLT3-L, and 25 mg/ml mouse IL-6 for 72 h, and then 4-OHT at the final concentration of 200 nM was added to the culture medium for 48 h. RNA was extracted from the cells and reverse-transcribed, followed by direct sequencing of cDNA of the mutant-introduced region of *Ddx41* transcript. We used the minimum number of animals that allowed to achieve statistical significance. No randomization was necessary. Due to the analysis in paired samples (cells treated with or without 4-OHT), no blinding

was necessary.

Statistical analysis. Statistical analysis was performed with R software (version 4.1.2). In conducting a *t* test for unpaired samples, we first performed an F-test with the significance level set at 0.05 to test whether data followed an equal distribution. Student's *t* test was used when data were believed to follow equal variances, and Welch's *t* test was used when data did not follow equal variances. In some experiments when indicated, a chi-square test was used. Sample sizes were chosen based on the degree of variability observed in similar experiments. All statistical details and sample sizes of experiments are given in corresponding figure legends, with p-values ≤ 0.01 being considered statistically significant unless otherwise indicated. Error bars in figures indicate SD. Inclusion/exclusion criteria were not applied, and there was no blinding. All experiments were repeated twice minimum in the laboratory.

Supplementary Figure S1

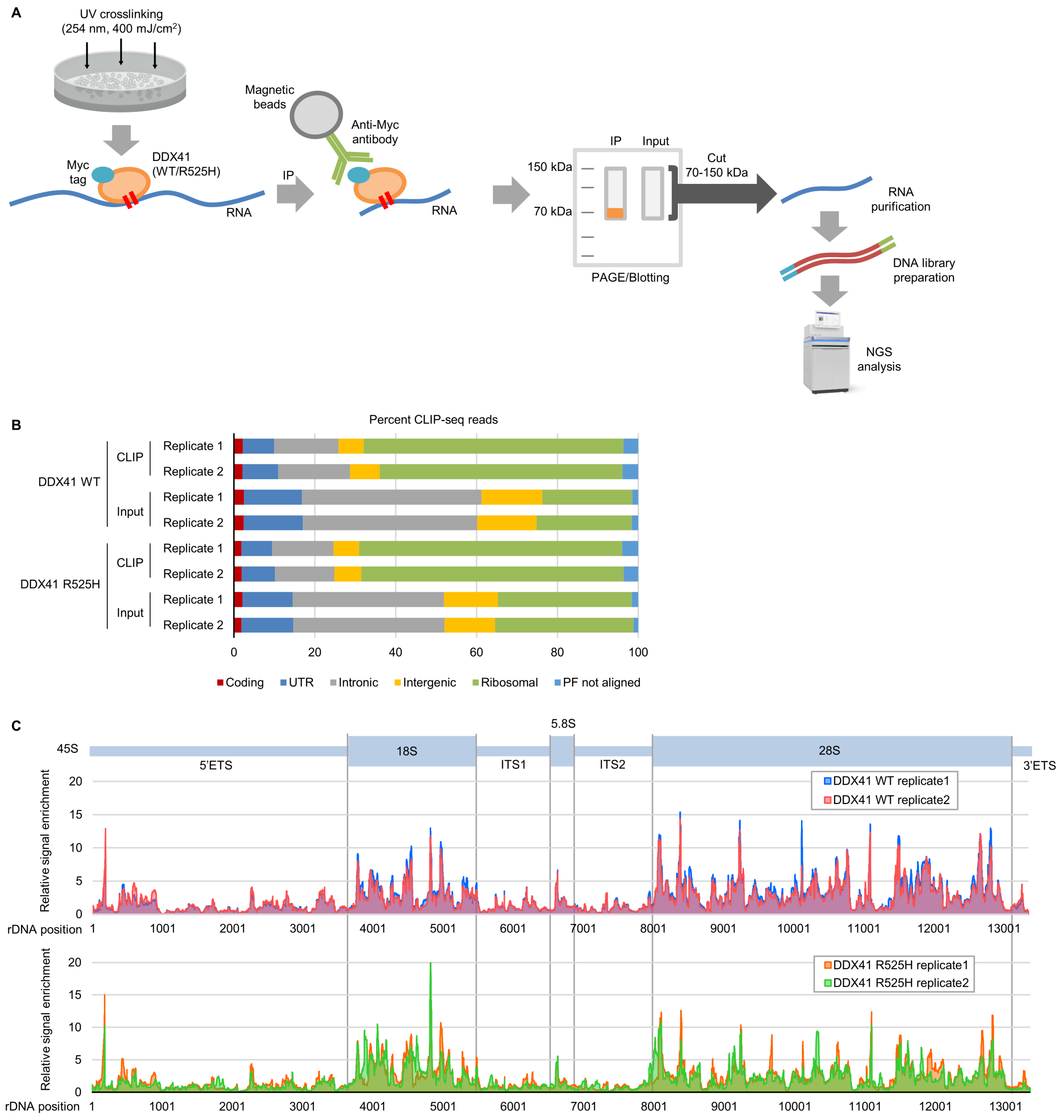

Supplementary Figure S1 (continued)

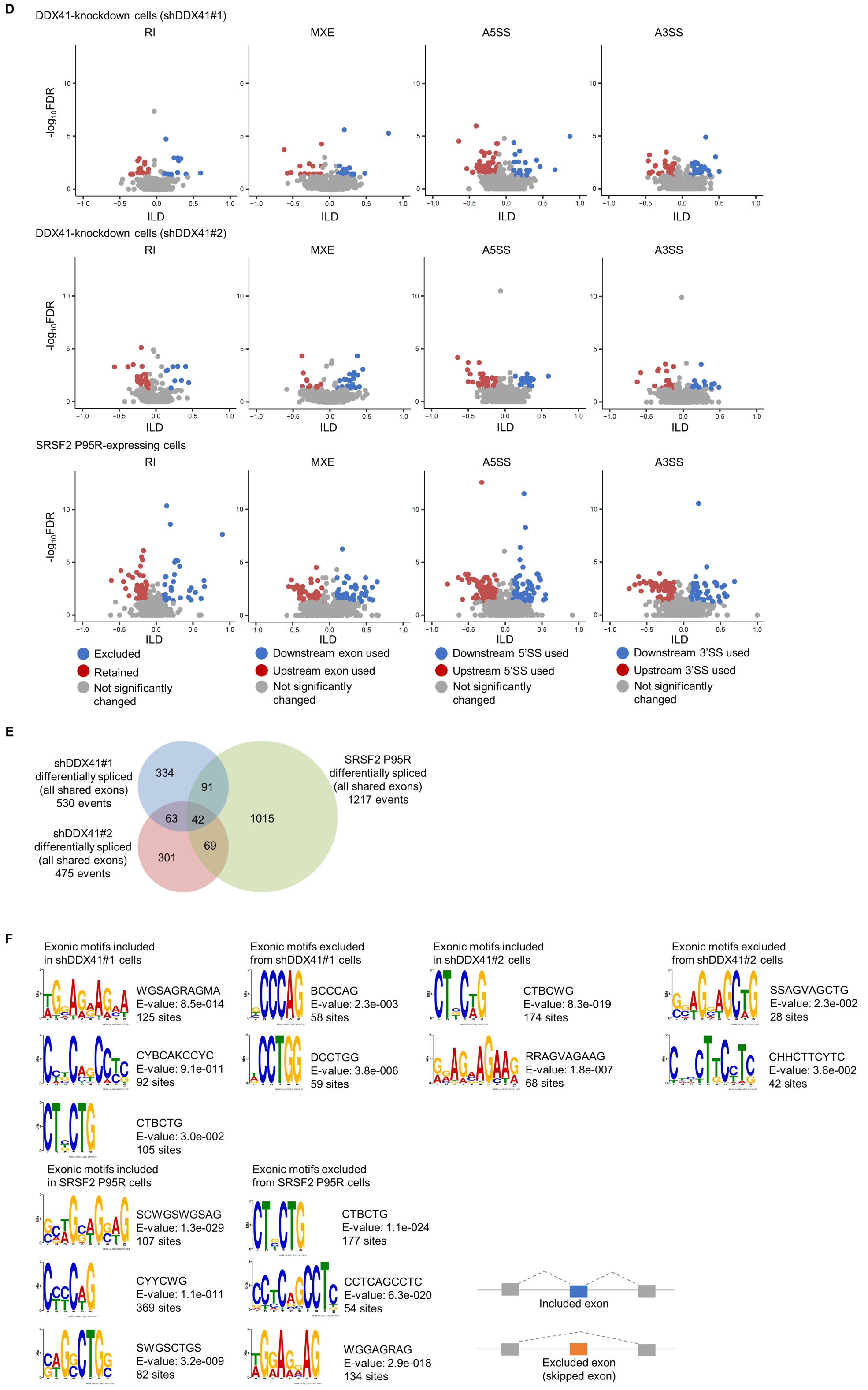

Supplementary Figure S1 (continued)

G

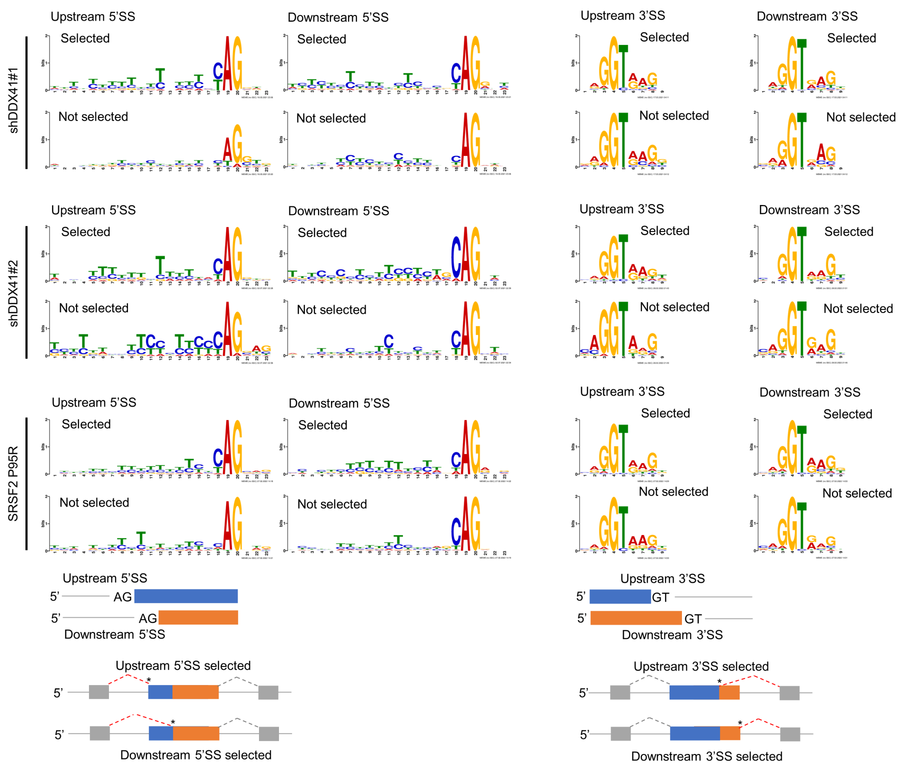

H

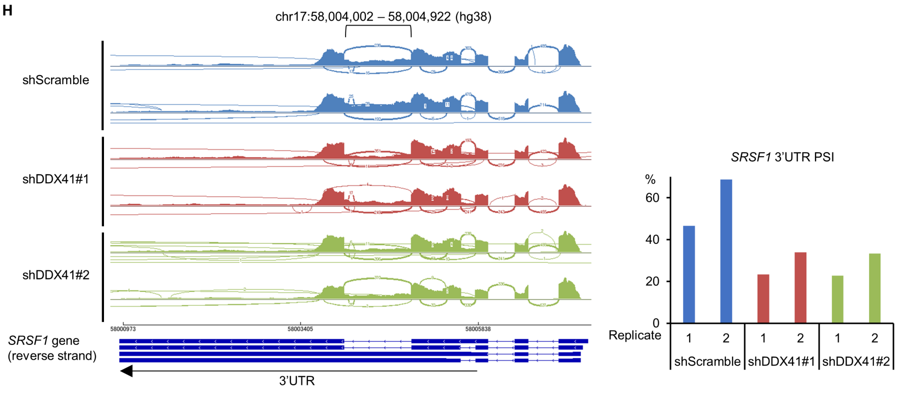

I

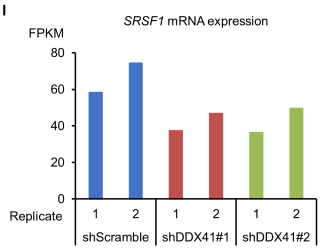

Supplementary Figure S1 (continued)

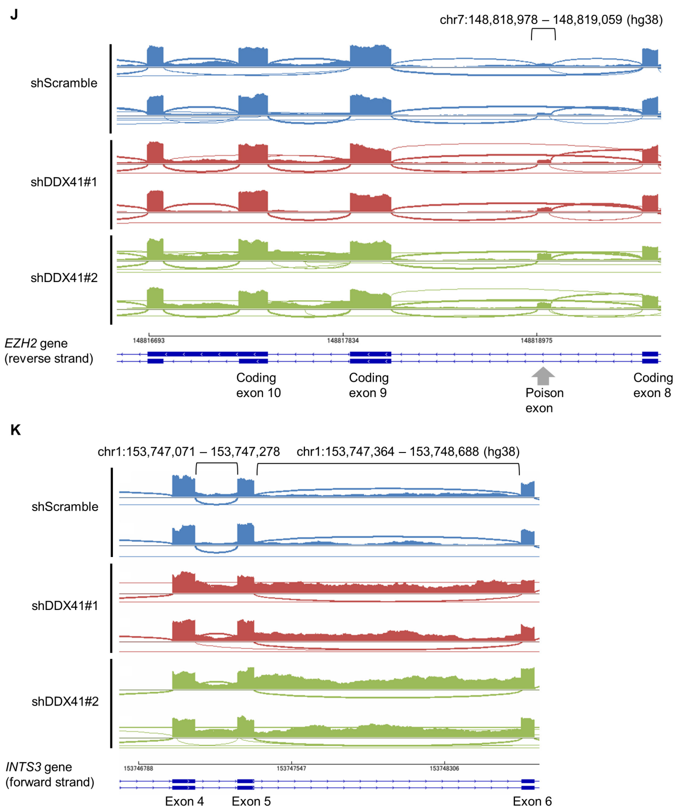

Supplementary Figure S1. RNA regions with which DDX41 interacts and splicing changes induced by DDX41 knockdown. (A) Schematic illustration of the CLIP-seq workflow for identification of RNA regions with which DDX41 interacts. K562 cells expressing Myc-tagged DDX41 (WT or R525H mutant) were crosslinked with UV light, and cell extracts were immunoprecipitated with an anti-Myc antibody. The immunoprecipitated samples were electrophoresed and transferred to a membrane, and the region containing DDX41 (70-150 kDa) was excised from the membrane. RNA was purified from the excised DDX41-RNA complex and processed to generate a sequencing

library. The libraries were sequenced by using a massive parallel next-generation sequencer (NGS). (B) RNA regions with which DDX41 interacts. RNAs extracted from CLIP-samples prepared by the method in (A) and input RNA were sequenced; sequenced reads were mapped to the human genome sequence (hg38) and sorted into genomic regions. PF, passing filter. (C) CLIP-seq reads mapped to the 45S rDNA region. The horizontal axis indicates positions in this region, and the vertical axis is the ratio of the CLIP sample signal divided by that of input RNA from the same cells. Blue and red lines in the top panel indicate relative signal enrichment of CLIP reads from cells expressing Myc-tagged WT DDX41; green and orange lines in the bottom panel indicate reads from cells expressing Myc-tagged R525H mutant DDX41. (D) Changes in RNA splicing events for RI, MXE, A5SS and A3SS in DDX41-knockdown cells and SRSF2 P95R-expressing cells. We included splicing events with 10% minimum change (delta PSI ≥ 0.1, see legend for Fig.1D) and average reads ≥5; those with FDR <0.05 with ILD <0.1 or >0.1 in each group were considered significant and plotted with red or blue dots, respectively. Gray dots are not significant. Splicing events were categorized as in Fig. 1B. (E) Overlap of shared RNA splicing events among DDX41-knockdown cells and SRSF2 P95R-expressing cells. All significant RNA splicing events (SE, MXE, RI, A5SS, and A3SS) that were expected to affect the same exon were summed for each cell type, and event overlap

among DDX41-knockdown cells and SRSF2 P95R-expressing cells is shown. (F) Motifs on exons with altered exon skipping in DDX41-knockdown cells and SRSF2 P95R-expressing cells. Exonic motifs included or excluded from mRNA in each cell type were analyzed via MEME, and significant motifs for each group are indicated in ascending order of E-values. Exon inclusion and skipped patterns are at the bottom right. (G) Sequence features around 5′SS or 3′SS with A5SS or A3SS in DDX41-knockdown cells and SRSF2 P95R-expressing cells. Sequence features of upstream and downstream splice sites used (selected) or not used (unselected) in mRNA of each cell type were analyzed and visualized via MEME. Meanings of upstream and downstream and selected and not selected are indicated at the bottom. Asterisks indicate alternative splice sites in each splicing event. (H) RNA splicing changes in the 3′ untranslated region of *SRSF1* in DDX41-knockdown cells. The chr17:58,004,002–58,004,922 in hg38 is a region that was identified by rMATS as more spliced in DDX41-knockdown cells (visualized by IGV in a Sashimi plot). The right-hand chart indicates PSI of the corresponding region. (I) *SRSF1* mRNA expression changes on DDX41 knockdown. *SRSF1* expression was quantified on the basis of mRNA-seq data and indicated as fragments per kilobase of transcript per million mapped reads (FPKM) values. (J, K) Increased usage of poison exon between coding exons 8 and 9 of *EZH2* and intron

retention between exons 4 and 6 of *INTS3* in DDX41-knockdown cells. Sashimi plots were generated and visualized with IGV, in which chr7:148,818,978–148,819,059 is the poison exon of *EZH2* gene that increased (J), and chr1:153,747,071–153,748,688 is the intronic regions in *INTS3* showing greater retention (K) in DDX41-knockdown cells.

Supplementary Figure S2

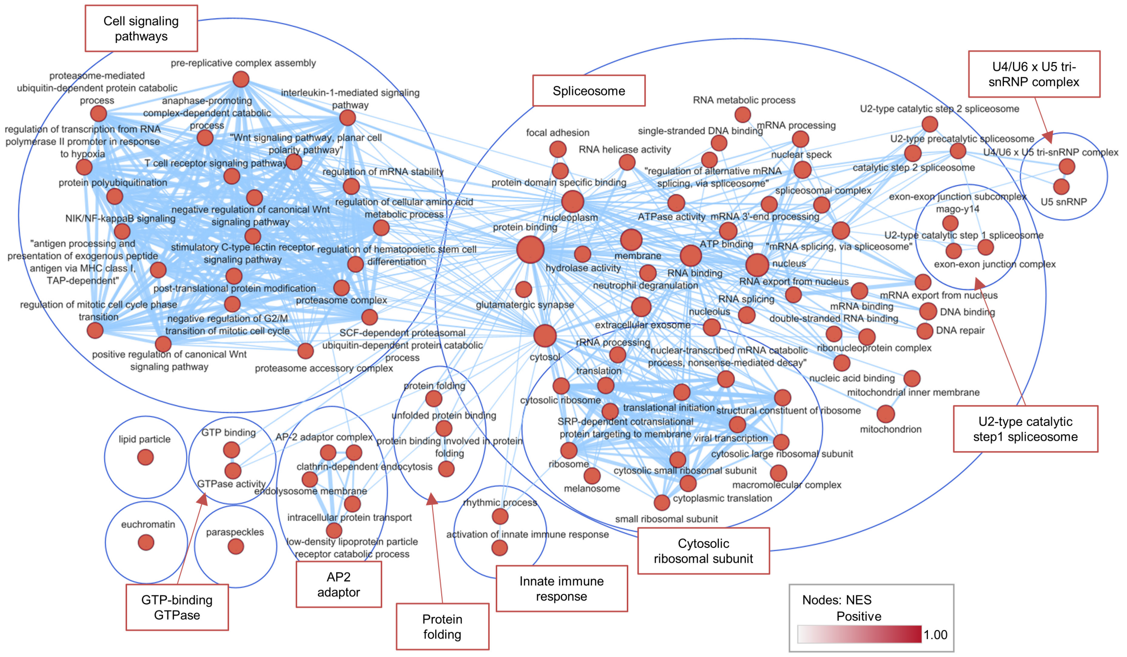

Supplementary Figure S2. Interaction of DDX41 with RNA splicing-related proteins. GO analysis of proteins immunoprecipitated with FLAG-DDX41. Nuclear proteins that were identified as specifically immunoprecipitated with FLAG-DDX41 were analyzed by using DAVID, and significantly enriched GO terms and their interrelationships were visualized via Cytoscape.

Supplementary Figure S3

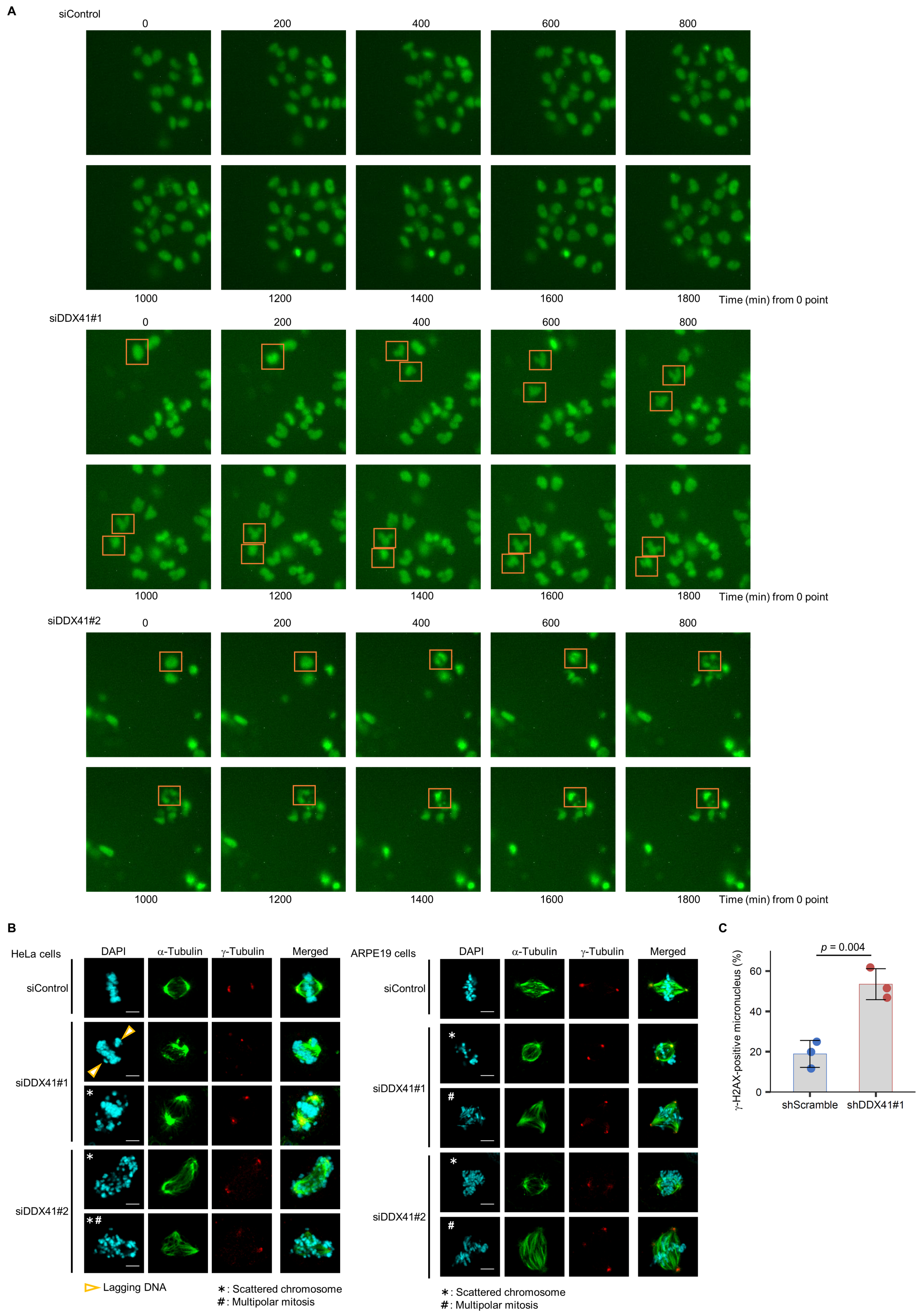

Supplementary Figure S3. Abnormal mitosis induced by DDX41 knockdown. (A) Abnormal nuclear morphology and mitosis induced by DDX41 knockdown. U2OS cells expressing GFP-tagged histone H2B were transfected with siRNA against DDX41. Time-lapse images were taken every 10 min with confocal microscopy, starting from 48 h after siRNA transfection (see Supplementary movies 1-3). Images in this figure were those cropped from the movie every 20 frames (200 min). Representative cells with abnormal nuclear morphology (siDDX41#1) or mitotic abnormalities (tripolar mitosis) (siDDX41#2) have orange squares to indicate changes over time. (B) Increased abnormal mitosis by DDX41 knockdown. Representative images of abnormal mitotic features observed in DDX41-knockdown HeLa (left) and ARPE19 (right) cells. Cells were transfected with siDDX41#1, siDDX41#2, or siControl. After 72 h, cells were stained for α-tubulin and γ-tubulin and were counterstained with DAPI. Scale bars: 5 μm. (C) Percent γ-H2AX-positive micronuclei in shScramble (n = 8, 15 and 17 micronuclei) and shDDX41#1 (n = 21, 31 and 32 micronuclei) groups was analyzed 3 days after lentivirus infection. Bars indicate means; error bars, SD of triplicate samples; two-tailed unpaired Student's t test.

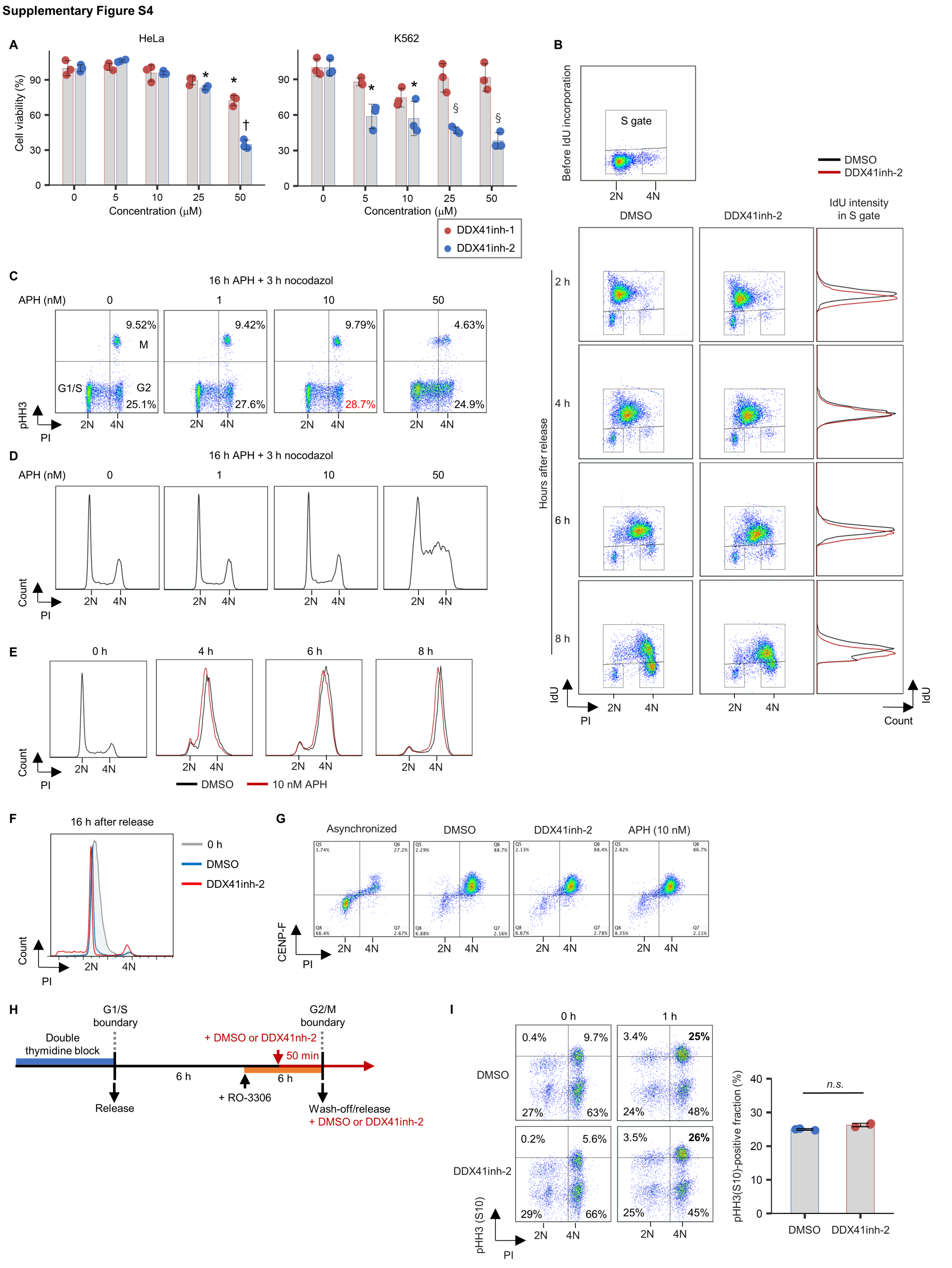

Supplementary Figure S4. Mild DNA replication stress in S phase by DDX41 inhibition.

(A) Dose-dependent inhibition of cell growth by DDX41 inhibitors. HeLa (left) and K562

(right) cells were treated with DDX41inh-1, DDX41inh-2, or DMSO for 78 h at the indicated concentrations, and relative cell numbers were determined. Bars indicate means; error bars represent SD of triplicate samples. *$p < 0.01$; †$p < 0.0001$; §$p < 0.0005$ vs. DMSO group; two-tailed unpaired Student's t test. (B) Reduced IdU incorporation and delayed S phase progression by DDX41 inhibition. The experiment was performed as indicated in Fig. 4C. (C, D) Changes in cell cycle distribution by APH. Asynchronized HeLa cells were treated with APH for 16 h at the indicated concentrations. For mitotic trap, cells were treated with 100 ng/ml nocodazole during the last 3 h of APH treatment. Cell cycle phases of each population are marked on the panels. Cells were stained with PI and anti-pHH3 (S10) antibody. Percentages of cells at G2 and M phases (C) and histograms of cell cycle profiles (D). (E) Delayed S phase progression by APH. HeLa cells were synchronized and treated with 10 nM APH or DMSO as indicated in Fig. 4C. Cells were stained with PI, and cell cycle distribution at indicated times after release from the G1/S boundary was measured by flow cytometry. (F) Completion of mitosis at 16 h after release from double thymidine block. HeLa cells were treated with DDX41inh-2 or DMSO as indicated in Fig. 4C. Cells were stained with PI, and cell cycle distribution at 16 h after release from the G1/S boundary was measured by flow cytometry. (G) Cell cycle arrest at G2 by RO-3306. HeLa cells were synchronized and treated with 50 μM

DDX41inh-2, 10 nM APH, or DMSO as indicated in Fig. 4I. Immediately before RO-3306 removal, cells were stained with PI and an antibody against CENP-F, a G2-phase marker. Cells at G2 are shown as CENP-F-positive 4N cells. (H) Schematic diagram of DDX41inh-2 treatment in G2 phase. HeLa cells were released for 6 h after double thymidine block, and RO-3306 was added to trap cells in G2 phase. Cells arrested at G2 were treated with 50 μM DDX41inh-2 or DMSO for 50 min and then released to allow cells to enter mitosis. (I) Induction of mitosis was not delayed when cells were treated with DDX41inh-2 in G2 phase. Cells were treated as shown in H and stained with PI and anti-pHH3 antibody. Cells at G2 or M phase were identified as those negative or positive for pHH3 with the PI signal corresponding to 4N, respectively. Proportion of pHH3-positive M phase cells determined 1 h after RO-3306 removal. Bars indicate means; error bars represent SD of triplicate samples; two-tailed unpaired Student's *t* test. *n.s.*, not significant.

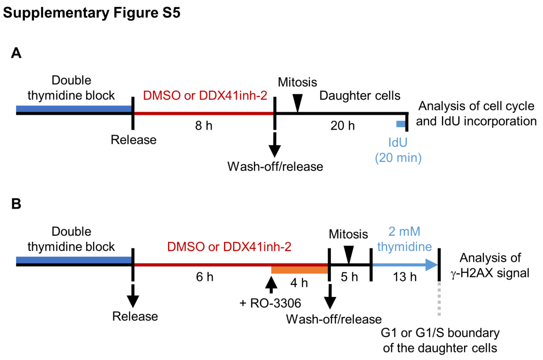

Supplementary Figure S5. Experimental workflow for cell cycle specific DDX41 inhibition. (A) Schematic diagram for analysis of cell cycle distribution and IdU incorporation of daughter cells subjected to DDX41 inhibition in S phase before mitosis. HeLa cells were released for 8 h after double thymidine block in the presence or absence of DDX41inh-2 before replacement of medium. After 20 h and labeling with IdU for the last 20 min, cells were stained with anti-BrdU antibodies and PI. Corresponding data are shown in Fig. 5B. (B) Schematic diagram for γ-H2AX analysis in daughter G1 cells. Five hours after release from G2 phase, cells were treated with thymidine for 13 h to trap daughter cells in G1 or the G1/S boundary and then stained with anti-γ-H2AX antibody and PI. Corresponding data are shown in Fig. 5E.

Supplementary Figure S6

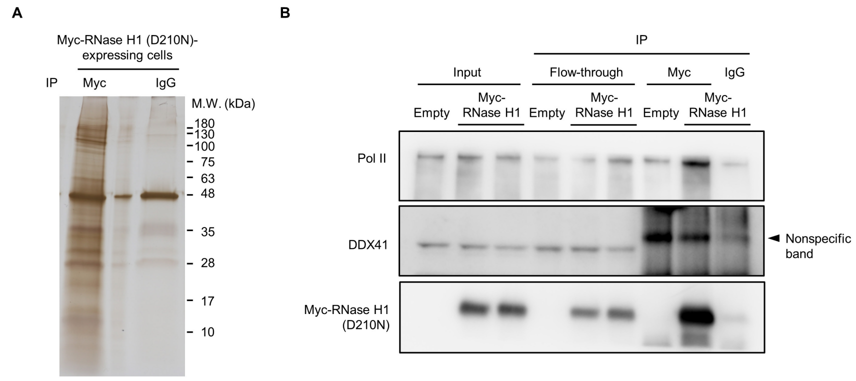

Supplementary Figure S6. Immunoprecipitation of R-loops and interacting proteins. Immunoprecipitation of R-loops with enzymatically inactive RNaseH1 expressed in the nucleus. HEK293FT cells were transiently transfected with Myc-tagged RNaseH1 mutant (D210N) that is engineered to lose enzymatic activity but retains R-loop-binding capacity. Cell extracts were immunoprecipitated with anti-Myc antibody or control IgG. Precipitated samples were electrophoresed and subjected to silver staining (A) or were probed with anti-Myc, anti-Pol II, or anti-DDX41 antibody (B).

Supplementary Figure S7

A

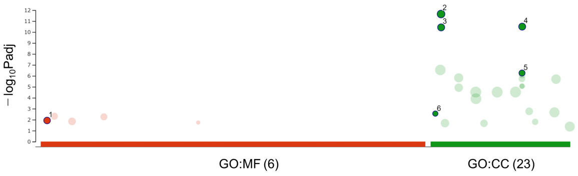

No.	Term Name	Term ID	$-\log_{10}$Padj
1	basal RNA polymerase II transcription machinery binding	GO:0001099	1.93
2	spliceosomal complex	GO:0005681	11.64
3	catalytic step 2 spliceosome	GO:0071013	10.49
4	U2-type spliceosomal complex	GO:0005684	10.42
5	U2-type catalytic step 2 spliceosome	GO:0071007	6.26
6	Prp19 complex	GO:0000974	2.55

B

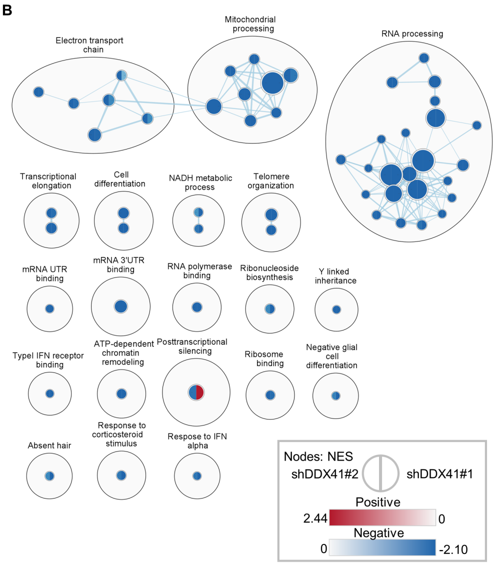

Supplementary Figure S7 (continued)

C

GO:0071013 Catalytic step 2 spliceosome

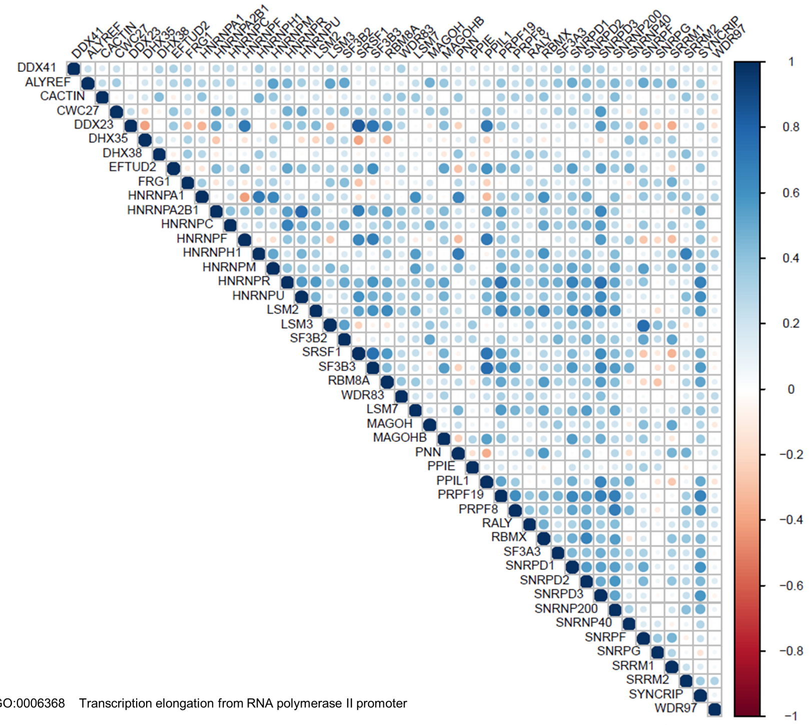

GO:0006368 Transcription elongation from RNA polymerase II promoter

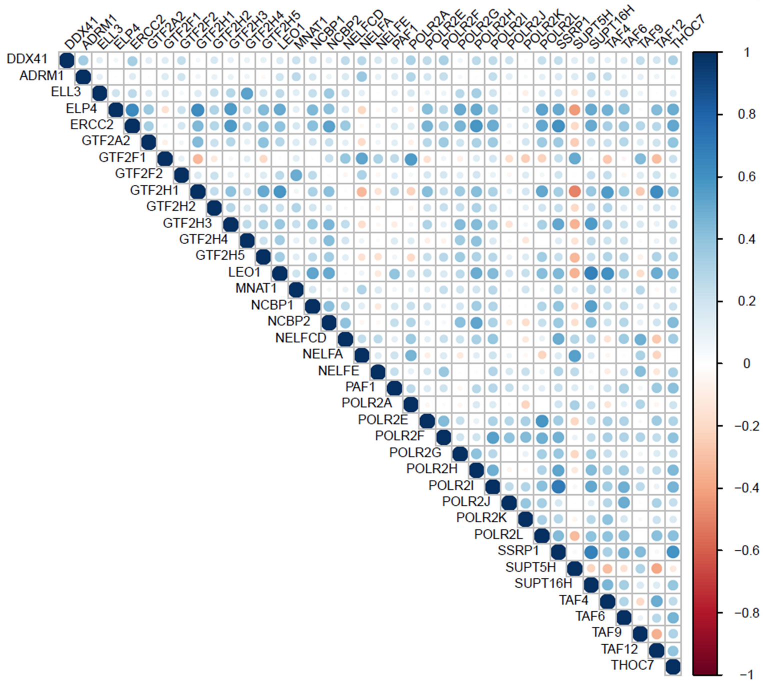

Supplementary Figure S7 (continued)

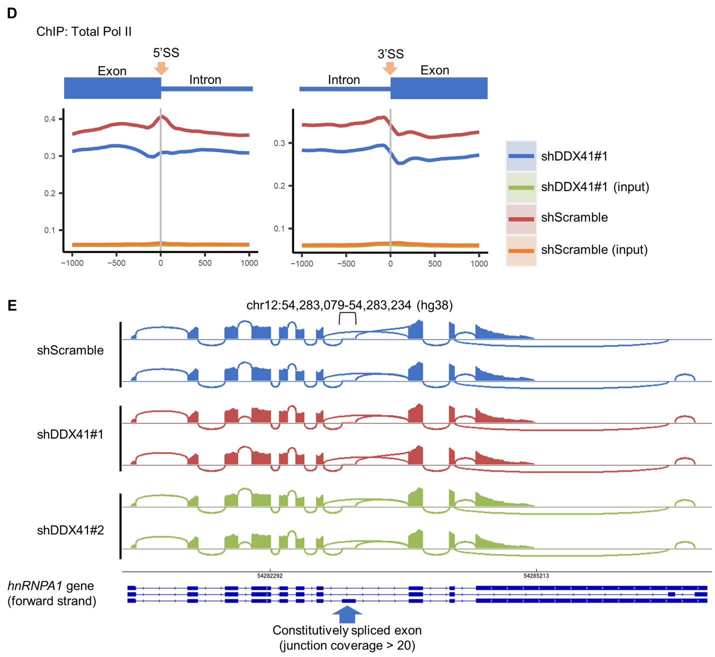

Supplementary Figure S7. DDX41 promotes transcriptional elongation by facilitating cooperation between the spliceosome and the transcriptional elongation complex. (A) Genes co-dependent with DDX41 include those related to RNA splicing and Pol II-mediated transcription. Top 100 co-dependent genes with DDX41 identified in RNAi screening were subjected to GO analysis, and results were visualized by using g:Profiler (upper panel). Representative GO terms related to RNA splicing (red) and Pol II-mediated transcription (blue) were numbered and are shown (lower). MF, molecular function. CC, cellular component. (B) GSEA of differently expressing genes between DDX41-knockdown cells and control cells. Gene sets positively or negatively enriched in shDDX41#1- and shDDX41#2-expressing cells were visualized by using Cytoscape. Node

colors represent the nominal enrichment score (NES) for each gene set, with blue and red nodes being gene sets negatively or positively enriched in DDX41-knockdown cells, respectively. (C) Interrelationships between expression levels of DDX41 and those related to the catalytic step 2 spliceosome and Pol II-mediated transcriptional elongation. Data in Fig. 7E was used. Correlations between DDX41 and factors included in step 2 spliceosome (GO: 0071013; upper) and transcription elongation from RNA polymerase II promoter (GO: 0006368; lower) are provided in a correlation matrix. Positive and negative correlations are indicated by blue and red circles, respectively, with circle colors indicating corresponding correlation coefficients. (D) Average distribution of Pol II around exon/intron boundaries when analyzed for exons expressed in DDX41-knockdown cells. Average Pol II distribution up to 1000 bases upstream and downstream of 5′SS and 3′SS were visualized by using Ngsplot as in Fig. 7I and J. Transcripts in RefSeq Select that were expressed above the median in shDDX41#1-expressing DDX41-knockdown cells were analyzed. (E) Representative image of the *hnRNPA1* gene with a constitutively spliced exon in K562 cells. Genes containing constitutively spliced exons were selected from those that were suggested to be skipped by analysis using rMATS, with criteria of coverage >20 and PSI <0.5. Arrow indicates an example of constitutive exon skipping in *hnRNPA1* gene.

Supplementary Figure S8

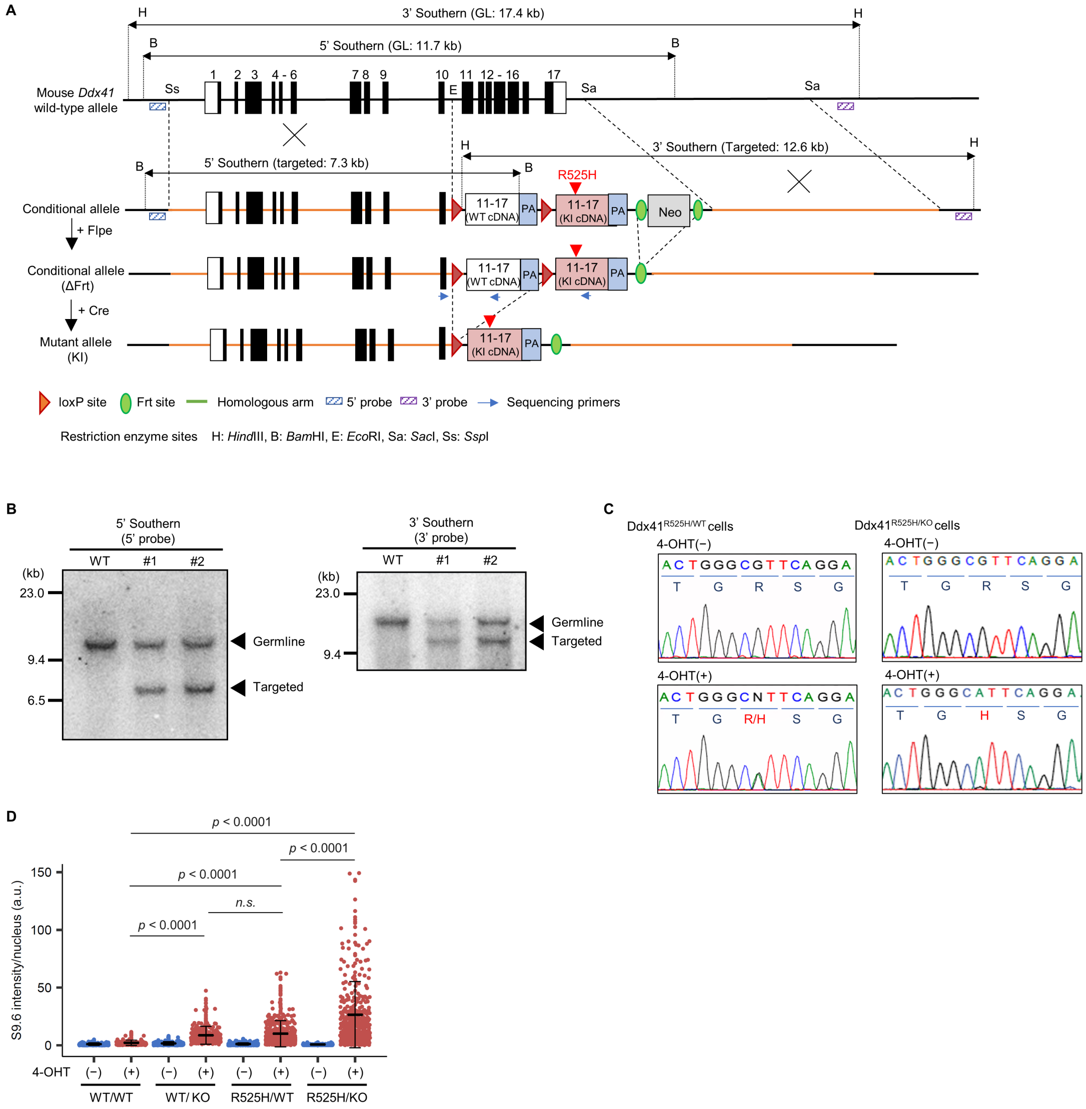

Supplementary Figure S8. DNA damage and R-loop accumulation after introducing R525H mutation in primary hematopoietic progenitor cells in mice. (A) Generation of the *Ddx41*^{R525H} conditional knock-in allele. Diagrams of the endogenous *Ddx41* locus, conditional allele, and modified alleles by Flpe and Cre activity. Numbers of boxes indicate exons and exonic sequences in cDNA; lines represent introns; PA indicates a

poly(A) transcriptional termination signal from SV40; Neo indicates the neomycin-resistance gene. The red box "11-17 (KI cDNA)" encodes cDNA of exon 11 to exon 17 carrying the R525H mutation. See Supplementary Methods for the detailed procedure to prepare the knock-in allele. (B) Confirmation of the introduction of the targeted allele. Southern blotting with the 5′ probe and 3′ probe to detect homologous recombination in two independent ES clones (#1 and #2). (C) Confirmation of induction of R525H mutation by 4-OHT treatment. Lin^{-}/c-Kit^{+} bone marrow cells isolated from *Ddx41*$^{WT/WT}$, *Ddx41*$^{WT/KO}$, *Ddx41*$^{R525H/WT}$ or *Ddx41*$^{R525H/KO}$ mice were cultured ex vivo in the presence of 25 ng/ml human TPO, 50 ng/ml mouse SCF, 50 ng/ml mouse FLT3-L, and 25 mg/ml mouse IL-6 for 72 h, and then 4-OHT at the final concentration of 200 nM was added to the culture medium for 48 h. RNA was extracted from cells and reverse transcribed, followed by direct sequencing of cDNA of the mutant-introduced region of the *Ddx41* transcript. Data from *Ddx41*$^{R525H/WT}$ and *Ddx41*$^{R525H/KO}$ cells are shown. (D) Increased R-loop formation in immature bone marrow cells from *Ddx41* heterozygous mice and those expressing R525H mutation. Cells cultured for 48 h in the presence or absence of 4-OHT were stained with S9.6 antibody. Quantitative signal levels are displayed for n = 201, 208, 258, 267, 317, 423, 191 and 343 for WT/WT/4-OHT^{-}, WT/WT/4-OHT^{+}, WT/KO/4-OHT^{-}, WT/KO/4-OHT^{+}, R525H/WT/4-OHT^{-}, R525H/WT/4-OHT^{+}, R525H/KO/4-OHT^{-},

and R525H/KO/4-OHT^{+}, respectively; two-tailed Welch's *t* test.

Supplementary Movies 1-3. Live monitoring of mitosis after DDX41 knockdown. Time-laps microscopy of GFP fluorescence in U2OS cells stably expressing GFP-tagged histone H2B transfected with siControl (movie 1), siDDX41#1 (movie 2) and siDDX41#2 (movie 3).

Supplementary References

1. Sarbassov DD, Guertin DA, Ali SM, Sabatini DM. Phosphorylation and regulation of Akt/PKB by the rictor-mTOR complex. Science. 2005;307:1098–101.

2. Jackson DA, Pombo A. Replicon clusters are stable units of chromosome structure: evidence that nuclear organization contributes to the efficient activation and propagation of S phase in human cells. J Cell Biol. 1998;140:1285–95.

3. Okuda H, Stanojevic B, Kanai A, Kawamura T, Takahashi S, Matsui H, et al. Cooperative gene activation by AF4 and DOT1L drives MLL-rearranged leukemia. J Clin Invest. 2017;127:1918–31.

4. Rubbi CP, Milner J. Analysis of nucleotide excision repair by detection of single-stranded DNA transients. Carcinogenesis. 2001;22:1789–96.

5. Kilgas S, Kiltie AE, Ramadan K. Immunofluorescence microscopy-based detection of ssDNA foci by BrdU in mammalian cells. STAR Protoc. 2021;2:100978.

6. Tyner JW, Tognon CE, Bottomly D, Wilmot B, Kurtz SE, Savage SL, et al. Functional genomic landscape of acute myeloid leukaemia. Nature. 2018;562:526–31.

7. Gao J, Aksoy BA, Dogrusoz U, Dresdner G, Gross B, Sumer SO, et al. Integrative analysis of complex cancer genomics and clinical profiles using the cBioPortal. Sci Signal. 2013;6:pl1.

8. Cerami E, Gao J, Dogrusoz U, Gross BE, Sumer SO, Aksoy BA, et al. The cBio cancer genomics portal: an open platform for exploring multidimensional cancer genomics data. Cancer Discov. 2012;2:401–4.

9. Tsherniak A, Vazquez F, Montgomery PG, Weir BA, Kryukov G, Cowley GS, et al. Defining a Cancer Dependency Map. Cell. 2017;170:564–76.

10. Raudvere U, Kolberg L, Kuzmin I, Arak T, Adler P, Peterson H, et al. g:Profiler: a web server for functional enrichment analysis and conversions of gene lists (2019 update). Nucleic Acids Res. 2019;47:W191–8.

11. Dobin A, Gingeras TR. Mapping RNA-seq Reads with STAR. Curr Protoc Bioinformatics. 2015;51:11.14.11–11.14.19.

12. Robinson JT, Thorvaldsdóttir H, Winckler W, Guttman M, Lander ES, Getz G, et al. Integrative genomics viewer. Nat Biotechnol. 2011;29:24–6.

13. Robinson MD, McCarthy DJ, Smyth GK. edgeR: a Bioconductor package for differential expression analysis of digital gene expression data. Bioinformatics. 2010;26:139–40.

14. Shen S, Park JW, Lu ZX, Lin L, Henry MD, Wu YN, et al. rMATS: robust and flexible detection of differential alternative splicing from replicate RNA-Seq data. Proc Natl Acad Sci U S A. 2014;111:E5593–601.

15. Bailey TL, Johnson J, Grant CE, Noble WS. The MEME Suite. Nucleic Acids Res. 2015;43:W39–49.

16. Huang da W, Sherman BT, Lempicki RA. Systematic and integrative analysis of large gene lists using DAVID bioinformatics resources. Nat Protoc. 2009;4:44–57.

17. Subramanian A, Tamayo P, Mootha VK, Mukherjee S, Ebert BL, Gillette MA, et al. Gene set enrichment analysis: a knowledge-based approach for interpreting genome-wide expression profiles. Proc Natl Acad Sci U S A. 2005;102:15545–50.

18. Otasek D, Morris JH, Bouças J, Pico AR, Demchak B. Cytoscape Automation: empowering workflow-based network analysis. Genome Biol. 2019;20:185.

19. Van Nostrand EL, Nguyen TB, Gelboin-Burkhart C, Wang R, Blue SM, Pratt GA, et al. Robust, Cost-Effective Profiling of RNA Binding Protein Targets with Single-end Enhanced Crosslinking and Immunoprecipitation (seCLIP). Methods Mol Biol. 2017;1648:177–200.

20. Van Nostrand EL, Pratt GA, Shishkin AA, Gelboin-Burkhart C, Fang MY, Sundararaman B, et al. Robust transcriptome-wide discovery of RNA-binding

protein binding sites with enhanced CLIP (eCLIP). Nat Methods. 2016;13:508–14.

21. Martin M. Cutadapt Removes Adapter Sequences From High-Throughput Sequencing Reads. EMBnetjournal. 2011;17:10–2.

22. Smith T, Heger A, Sudbery I. UMI-tools: modeling sequencing errors in Unique Molecular Identifiers to improve quantification accuracy. Genome Res. 2017;27:491–9.

23. Ewels P, Magnusson M, Lundin S, Käller M. MultiQC: summarize analysis results for multiple tools and samples in a single report. Bioinformatics. 2016;32:3047–8.

24. Miyamoto R, Yokoyama A. Protocol for fractionation-assisted native ChIP (fanChIP) to capture protein-protein/DNA interactions on chromatin. STAR Protoc. 2021;2:100404.

25. Langmead B, Salzberg SL. Fast gapped-read alignment with Bowtie 2. Nat Methods. 2012;9:357–9.

26. Shen L, Shao N, Liu X, Nestler E. ngs.plot: Quick mining and visualization of next-generation sequencing data by integrating genomic databases. BMC Genomics. 2014;15:284.
